# The Arabidopsis Receptor Kinase ZAR1 Is Required for Zygote Asymmetric Division and Its Daughter Cell Fate

**DOI:** 10.1371/journal.pgen.1005933

**Published:** 2016-03-25

**Authors:** Tian-Ying Yu, Dong-Qiao Shi, Peng-Fei Jia, Jun Tang, Hong-Ju Li, Jie Liu, Wei-Cai Yang

**Affiliations:** State Key Laboratory of Molecular Developmental Biology, National Center for Plant Gene Research (Beijing), Institute of Genetics and Developmental Biology, Chinese Academy of Sciences, Beijing, China; The University of North Carolina at Chapel Hill, UNITED STATES

## Abstract

Asymmetric division of zygote is critical for pattern formation during early embryogenesis in plants and animals. It requires integration of the intrinsic and extrinsic cues prior to and/or after fertilization. How these cues are translated into developmental signals is poorly understood. Here through genetic screen for mutations affecting early embryogenesis, we identified an Arabidopsis mutant, *zygotic arrest 1 (zar1)*, in which zygote asymmetric division and the cell fate of its daughter cells were impaired. *ZAR1* encodes a member of the RLK/Pelle kinase family. We demonstrated that ZAR1 physically interacts with Calmodulin and the heterotrimeric G protein Gβ, and ZAR1 kinase is activated by their binding as well. *ZAR1* is specifically expressed micropylarly in the embryo sac at eight-nucleate stage and then in central cell, egg cell and synergids in the mature embryo sac. After fertilization, ZAR1 is accumulated in zygote and endosperm. The disruption of *ZAR1* and *AGB1* results in short basal cell and an apical cell with basal cell fate. These data suggest that ZAR1 functions as a membrane integrator for extrinsic cues, Ca^2+^ signal and G protein signaling to regulate the division of zygote and the cell fate of its daughter cells in Arabidopsis.

## Author Summary

Flowering plants are featured as double fertilization, a process that the egg cell and the central cell of embryo sac fuse with a sperm and give rise to a diploid zygote and a triploid primary endosperm cell, respectively. The zygote develops into embryo after cell division and differentiation, and starts a new trip of next generation. Meanwhile, the primary endosperm cell proceeds nuclear division to generate a syncytium and develops into endosperm after cellularization. Embryo development initiates from asymmetric division of zygote. A small apical cell and a long basal cell are produced after the first zygotic division, which establishes the pattern of an early embryo. To unveil the molecular mechanism controlling zygote asymmetric division, we screened our *Ac/Ds* insertion lines for mutations controlling early embryogenesis, one of the mutations *zygotic arrest 1* (*zar1*) was reported here. In *zar1*, zygote was either arrested after elongation or displayed symmetric division. The mutation also had a slight impact on the apical- and basal-cell fates manifested by the mis-expression of the cell-lineage specific markers. *ZAR1* encodes a member of the RLK/Pelle kinase family, and interacts physically with Calmodulin and the heterotrimeric G protein Gβ, both *in vitro* and *in vivo*. These data suggest that ZAR1 might act as an integrator for intracellular Ca^2+^ and heterotrimeric G protein signaling with extracellular signals during early zygote development. Interestingly, complete loss of Gβ or ZAR1 function displayed very weak phenotype. This suggests that there might be genetic redundancy and plasticity during early embryogenesis. More studies are needed to dissect the complexity of early embryo development in plants.

## Introduction

Asymmetric cell division is a fundamental process which produces daughter cells with different size and cell fate during embryonic and postembryonic development. Irrespective of cell types or organisms, it requires a common set of coordinated events including the establishment and transduction of polarity, and cytokinesis [1–3]. Cell polarity can be set up by intrinsic or/and extrinsic factors [4]. During early embryogenesis, the zygotic polarity is established either by maternal determinants prior to fertilization in Drosophila, or by sperm entry at fertilization in *Caenorhabditis elegans*. In higher plants, studies have been largely focused on easily accessible epidermal and root cells [5, 6], little is known about molecular mechanisms underlying the asymmetric division of zygote. In Arabidopsis, a YODA-mediated MAPK pathway was shown to be critical for asymmetric cell division in stomata and zygote development [5, 7–9]. So far, the BASL-MAPK signaling feedback was shown to control stomata asymmetric division. The phosphorylation of BASL (BREAKING OF ASYMMETRY IN THE STOMATAL LINEAGE) mediated by MPK3/6, is required for the cortical localization of BASL. YODA and MPK3/6 are subsequently recruited by the phosphorylated BASL. The feedback loop is further promoted by MPK3/6. It is demonstrated that cell polarity and fate determination are reinforced and connected by a positive feedback loop of BASL-MAPK during asymmetric division [8, 9]. *YODA* encodes a MAPKK kinase that promotes zygote elongation and the basal extra-embryonic cell fate [10]. The MAPKK kinase cascade, on the other hand, is likely activated by the paternal SHORT SUSPENSOR (SSP) [11, 12]. However, the kinase activity of SSP is not required for YODA activation. A small nuclear protein, GROUNDED (GRD), is also required for zygote elongation and the first asymmetric division to establish the basal cell fate [7, 13]. Recently, it was reported that EMBRYO SURROUNDING FACTOR 1 (ESF1) peptides from central cell before fertilization act with SSP to promote suspensor elongation through the YODA pathway [14]. These suggest that the conserved MAPK cascade plays a key role in zygote asymmetric division and basal cell fate determination. In addition, *WOX* (*Wuschel*-*related homeobox*) genes also play critical roles during early embryogenesis and serve as cell fate determinants of the apical and basal cell lineages [15, 16]. *WOX* genes, on the other hand, are directly activated by other transcription factors like WRKY2 [17]. In general, extracellular stimuli are received by membrane receptor kinases, and subsequently integrated and transduced inward via numerous signaling molecules [18]. Question remains to be elucidated that how the extracellular stimuli are perceived during early embryogenesis, and how the receptor kinases activate downstream MAPK signaling cascade need to be identified, too.

To gain insights into molecular mechanisms controlling zygote development, a detailed screen of our *Ds* insertion collections for mutations affecting early embryogenesis was performed [19]. A *Ds* insertion mutant, *zygotic arrest 1* (*zar1)*, whose zygote elongates normally but fails to perform asymmetric division, was identified. Furthermore, cell fate specification of both apical and basal cells is affected by the mutation manifested by the mis-expression of cell-specific markers. The enforcement of the basal and apical cell fate is likely dependent on ZAR1 and AGB1 functions. *ZAR1* encodes a leucine-rich repeat receptor-like kinase (LRR-RLK) that contains a putative CaM-binding domain and a Gβ-binding motif within its intracellular kinase region. Our data indicate that ZAR1 kinase activity is activated through its direct interaction with CaM1 and the heterotrimeric G protein Gβ (AGB1). We hypothesize that ZAR1 integrates extracellular stimuli with intracellular Ca^2+^ and G-protein signaling, to modulate zygotic division in Arabidopsis.

## Results

### Zygote division is impaired in *zar1* mutants

**Fig 1:**
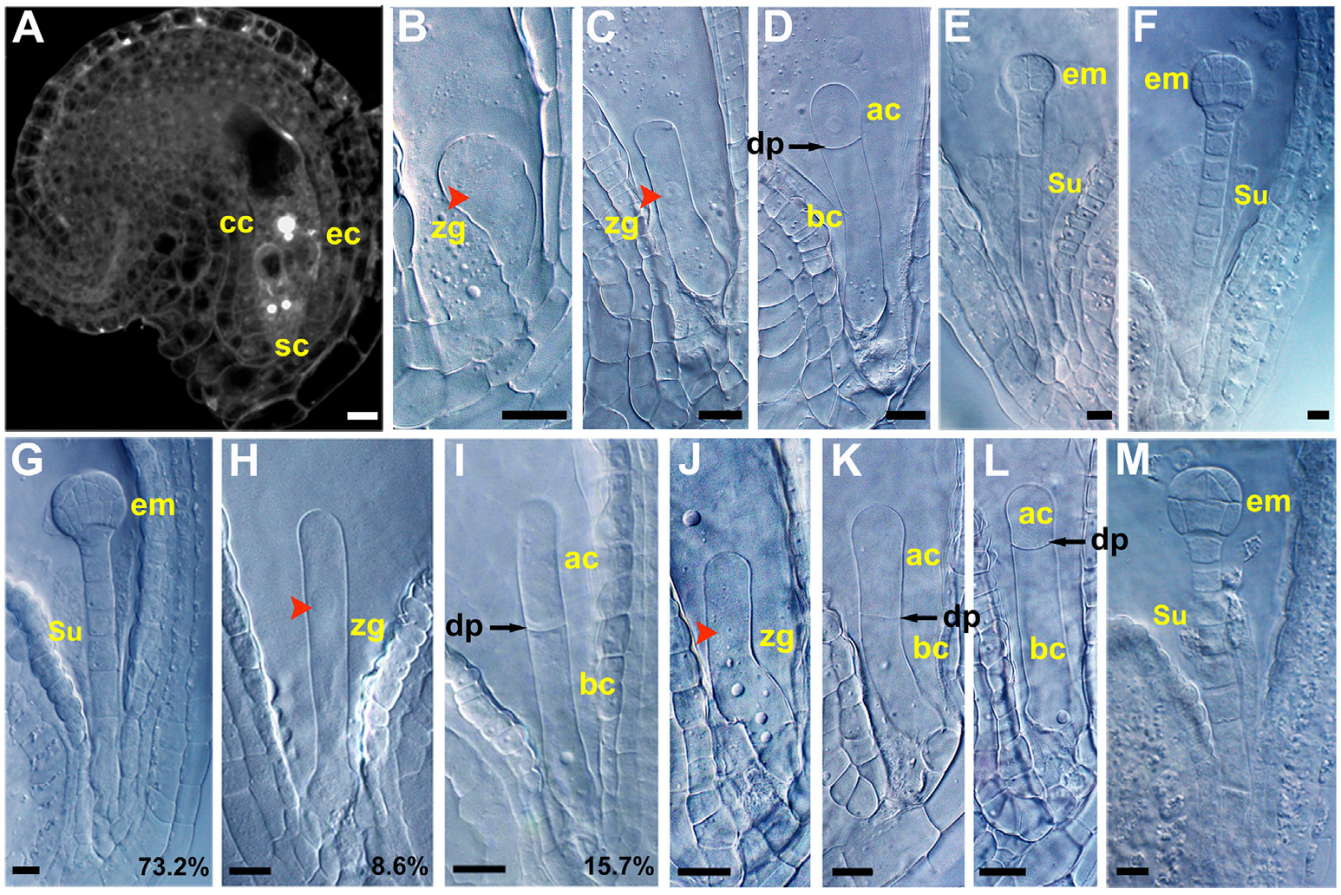
Early embryo development in the wild-type plants and *zar* mutants. (**A**) A mature wild-type embryo sac showing the egg cell (ec), central cell (cc) and synergid cells (sc) under confocal microscopy. (**B-F**) Early embryo in wild type showing zygote (zy) (**B**), elongated zygote (**C**), the first zygotic division to produce a small apical cell (ac) and a large basal cell (bc) (**D**), early globular embryo (em) (**E**), and globular embryo (**F**). (**G-I**) Early embryogenesis in *zar1-1*^*+/-*^ seeds showing normal globular embryo (**G**), elongated and arrested zygote (**H**), and symmetrically divided zygote (**I**). (**J-M**) early embryogenesis in *zar1-2* seeds showing elongated zygote (**J**), symmetric division of zygote (**K**), asymmetrically divided zygote (**L**), and globular embryo (**M**). dp, division plane; su, suspensor; red arrowheads indicate nuclei. Bars = 10 μm.

Double fertilization is a unique reproductive process of flowering plants, in which two female gametes (the egg and the central cell) in the embryo sac (Fig 1A) fuse with two male gametes (the sperms), to produce zygote ((Fig 1B) and endosperm, respectively. Following a quiescent stage after fertilization, the zygote undergoes a series of morphological changes that lead to the establishment of zygote polarity and the zygote elongates to about three folds along the future apical-basal embryo axis (Fig 1B and 1C). Subsequently, an asymmetric division occurs and a small apical cell which gives rise to the embryo proper, and a large basal cell that forms the suspensor connecting the embryo and the mother tissue, are produced (Fig 1D–1F). The uppermost cell of the basal lineage forms the hypophysis that is ultimately incorporated into the embryonic root. This stereotyped development in Arabidopsis serves as a model for genetic dissection of early embryo development in flowering plants [20, 21].

**Fig 2:**
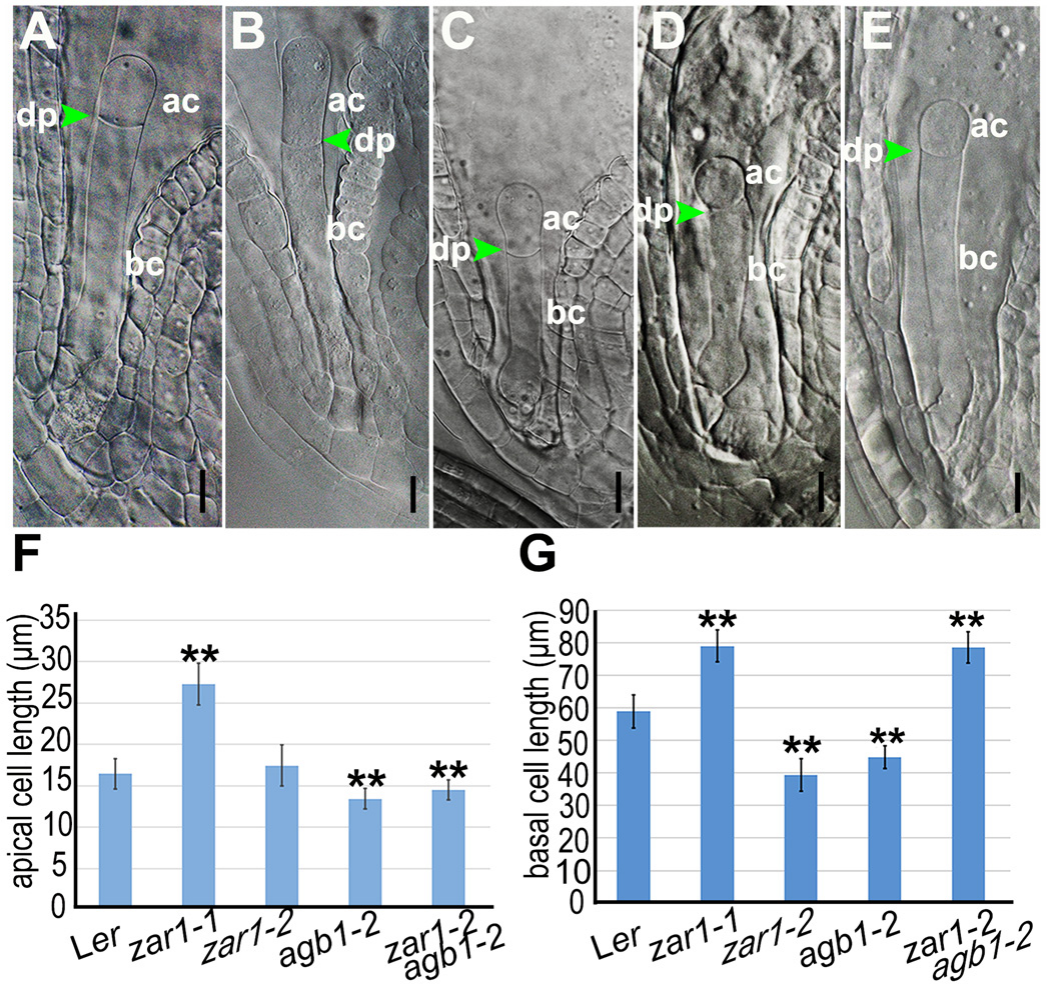
Zygotic phenotype of *zar1* and *agb1* mutations. Microscopic images showing the size of apical cell and basal cell in the wild-type L*er* (**A**), *zar1-1* (B), *zar1-2* (**C**), *agb1-2* (**D**), and *zar1-2 agb1-2* (**E**) mutants. Statistical results on apical cell (**F**) and basal cell (**G**) are shown (n > 30). ac, apical cell; bc, basal cell; dp, division plane. Stars indicate significant difference. Bars = 10 μm.

In *zar1-1*^*+/-*^ (for the simplicity, the heterozygous mutant is marked as z*ar1-1*^*+/-*^ and the homozygous as z*ar1-1* or *zar1-1*^*-/-*^) siliques, three days after pollination (DAP), 73.2±0.34% (n = 1286) ovules contain early globular embryo as in the wild-type (Fig 1G), while the other ovules contain either an elongated zygote with an obvious nucleus almost localized at the central (8.6±1.5%, n = 1286) (Fig 1H) or two similar cells from a nearly symmetric division (15.7±1.9%, n = 1286) (Fig 1I). In total, they account for about 26.8% (n = 1286) of the ovules. This indicates that the mutation does not affect zygote elongation but hinders it from division or disrupts the first asymmetric division. These ovules are also defective in endosperm development (S1 Fig). Genetic analysis showed that the *Kan*^*R*^/*Kan*^*S*^ ratio in selfed *zar1-1*^*+/-*^ progenies is 1.8:1 (n = 1038), close to the ratio 2:1 (χ^2^ = 1.35) for embryo lethal mutation; the *Kan*^*R*^*/Kan*^*S*^ is 1:1.03 (n = 788) when *zar1-1*^*+/-*^ was used as male or 1:1.19 (n = 879) as female in crosses with the wild-type. These indicate that homozygous *zar1-1*^*-/-*^ is embryo lethal, but male or female gametogenesis is not impaired by the mutation. The ratio of the normal to abnormal ovules is 2.7:1, in agreement with a typical recessive mutation of 3:1 segregation (P < 0.05, χ^2^_0.05_ = 3.84). Meanwhile, we obtained two weak alleles, *zar1-2* (a *Ds* insertion line) and *zar1-3* (a *T*-*DNA* mutant). Both *zar1-2* and *zar1-3* are homozygous with very similar phenotype. In the *zar1-2* siliques at 3 DAP, the seed set rate is 98±2.02% (n = 526). However, about 19.41±0.52% (n = 328) zygotes undergo symmetric, or approximately symmetric division in *zar1-2* siliques (Fig 1J and 1K), although other zygotes undergo asymmetric division (Fig 1L) as the wild-type. Statistical analysis indicates that the length of *zar1-2* basal cell (39.33±5 μm, n > 30) is significantly reduced compared to the wild-type (58.9±5.1 μm, n > 30) (see Fig 2A, 2C and 2G), because of an approximately symmetric division of zygote. Nevertheless, an early globular embryo is observed in every ovule two days later (Fig 1M), suggesting that there is an endurable disruption during early embryogenesis. Together, these indicate that *ZAR1* plays a critical role in zygote asymmetric division, and genetic redundancy exists for ZAR1 function.

### The *zar1* mutation modulates embryonic cell fate

**Fig 3:**
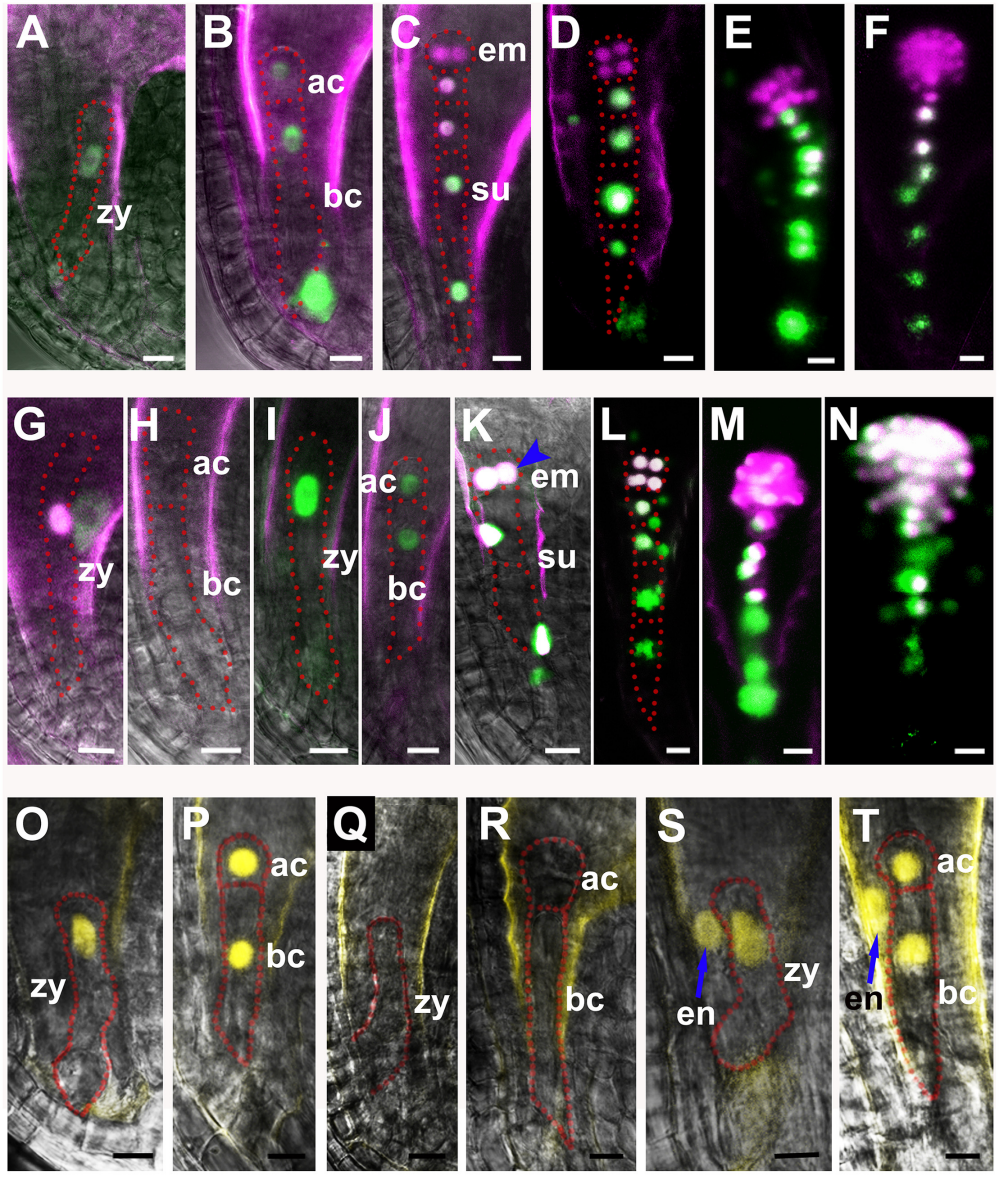
Ectopic expression of cell-specific markers in mutant embryo. (**A-N**) Basal cell-specific marker *pWOX8gΔ*:*NLS-vYFP*_*3*_ and apical cell-specific marker *pWOX2*:*DsRed2* are expressed in the wild-type (**A-F**), *zar1-1* (**G-H**), and *zar1-2* embryos (**I-N**). YFP-NLS signal of *pWOX8gΔ*:*NLS-vYFP*_*3*_ is only detected in zygote (**A**) and the basal cell lineage (**B-F**) in the wild-type, while in arrested *zar1-1* zygote (**G**), very weak YFP signal is present, but no YFP in apical cell, or basal cell (**H**). The *WOX8* is falsely expressed in the apical lineage besides the basal cell lineage in *zar1-2* embryo (blue arrowhead) (**K-N**). And apical cell-specific marker *pWOX2*:*DsRed2* is mis-expressed in arrested *zar1-1* zygote (**G**). YFP signal is falsely colored with green and DsRed signal with purple. (**O-T**) Expression of zygote asymmetric division marker *pWRKY2*:*NLS-vYFP*_*3*_ in wild type (**O-P**), arrested *zar1-1* seeds (**Q-R**) and *zar1-2* ovules (**S-T**). Blue arrows show the ectopic expression of *pWRKY2*: *NLS- vYFP*_*3*_ in endosperm of *zar1-2* ovules. ac, apical cell; bc, basal cell; dp, division plane; em, embryo; su, suspensor; zy, zygote. Bars = 10 μm.

To test whether the mutation affects the cell fate of the apical and basal cell lineages, we checked the expression of a basal cell-specific marker *pWOX8gΔ*:*NLS-vYFP*_*3*_ [15] and an apical cell-specific marker *pWOX2*:*DsRed2* [17]. As previously reported in wild-type plants, the YFP is only detected in zygote and the basal cell lineage, but absent in the apical cell lineage of *pWOX8gΔ*:*NLS-vYFP*_*3*_ transgenic plants (Fig 3A–3F). The apical cell lineage marker DsRed signal is detected in zygote, apical cell, the apical cell lineage and cells adjacent to embryo proper from basal cell lineage (Fig 3A–3F). In *zar1-1* arrested ovules, very weak YFP signal was detected in elongated zygote, but no YFP was observed in apical cell, or basal cell (Fig 3G and 3H, S2I–S2L Fig), indicating that the identity of these cells is impaired in *zar1-1* plants. In *zar1-2*, however, the YFP signal was strong in zygote (Fig 3I) and both the apical and basal cells (Fig 3J) as in the wild-type (Fig 3A and 3B), and unexpectedly present in the embryo proper besides the basal lineage (Fig 3K–3N, S2 Table) as compared to the wild-type (Fig 3C–3F). In comparison, the DsRed was only detected in elongated zygote (Fig 3G) but not in the apical or basal cells in *zar1-1* arrested ovules (Fig 3H). We further analyzed a *trans*-heterozygote made with *zar1-1*^*+/-*^/*pWOX8gΔ*:*NLS-vYFP*_*3*_/*pWOX2*:*DsRed2* pollinated with *zar1-2*^*-/-*^/*pWOX8gΔ*:*NLS-vYFP*_*3*_/*pWOX2*:*DsRed2*pollen grains and checked the expression of *pWOX8gΔ*:*NLS-vYFP*_*3*_/*pWOX2*:*DsRed2* in zygote and proembryo, we found that in about half seeds (n = 142), the expression pattern of *WOX2* and *WOX8* is consistent with the signal in *zar1-1* (S2A–S2D Fig), however, the other half seeds are similar to the wild type. This analysis showed that the *pWOX8gΔ*:*NLS-vYFP*_*3*_ and *pWOX2*:*DsRed2* markers are mis-expressed in arrested zygotes and daughter cells from the symmetrical division in *zar1-1*, indicating the *zar1-1* mutation impairs zygote and the apical/basal cell fate. The ectopic expression of the *pWOX8gΔ*:*NLS-vYFP*_*3*_ marker in the apical lineage during early embryogenesis in *zar1-2* suggests that the *zar1-2* mutation affects the apical and basal cell lineages.

Similarly, we checked the expression of *pWRKY2*:*NLS-vYFP*_*3*_ [16]. The YFP signal is found in zygote, the apical cell and the basal cell in wild-type ovules (Fig 3O and 3P); but no signal is detected in these cells in *zar1-1* arrested ovules (Fig 3Q and 3R); conversely, expression of *WRKY2* is detected ectopically in endosperm besides zygote and its daughter cells in *zar1-2* ovules (Fig 3S and 3T). This shows that *WRKY2* expression is also impaired in *zar1* mutants. Taking together, we concluded that the mutations of *ZAR1* have an effect on the cell fate specification of both the apical and basal cell lineages during early embryogenesis in Arabidopsis.

### *ZAR1* encodes a plasma membrane receptor kinase

**Fig 4:**
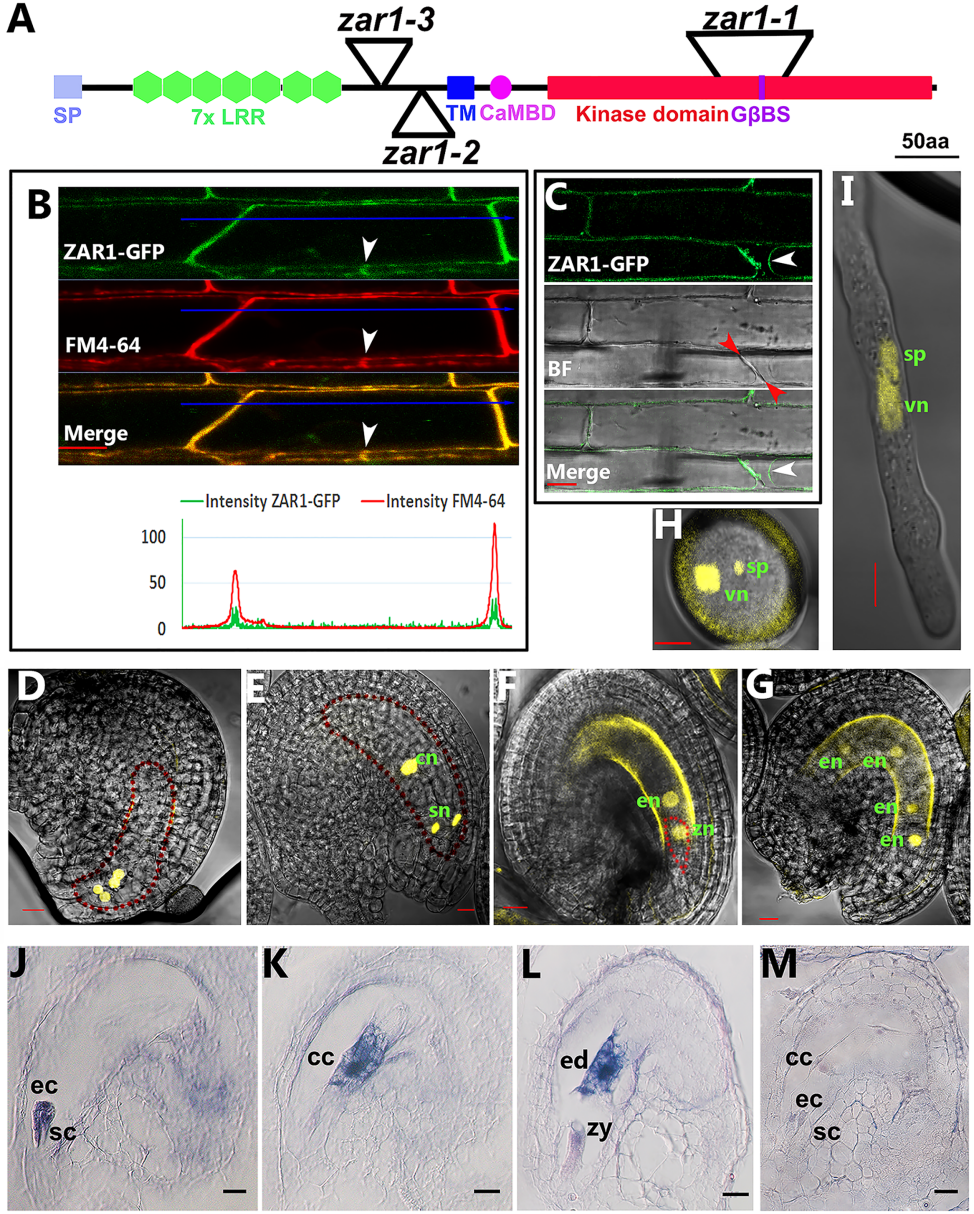
ZAR1 localization and expression pattern in embryo sac and early embryogenesis. (**A**) A catoon depicting the domains of ZAR1 protein and insertion sites in alleles *zar1-1*, *zar1-2* and *zar1-3*. SP, signal peptide; LRR, leucine-rich repeat; TM, transmembrane domain; CaMBD, calmodulin-binding domain; GβBS, Gβ binding site. (**B**) Co-localization of ZAR1-GFP with FM4-64 membrane probe in root cells over-expressing *ZAR1-GFP*. Plasmodesma was shown with white arrowhead. The bottom chart shows the profiling data of ZAR1-GFP and FM4-64. Bar = 5 μm. (**C**) Plasmolysis of root cells over-expressing *ZAR1-GFP*. Plasma membrane and cell wall were shown with white and red arrowheads, respectively. Bar = 10 μm. (**D-L**) Expression pattern of *ZAR1*. YFP signal from *pZAR1*:*NLS-YFP* transgenic plants is detected in the micropylar nuclei of eight-nucleate embryo sac (**D**), and in syngerid and central cells in mature embryo sac (**E**), and also in zygote (**F**) and endosperm after pollination (**F, G**); YFP is also detected in sperm and vegetative cells of both pollen grain (**H**) and pollen tube (**I**). Serial sections of the wild-type ovules were hybridized to *ZAR1* antisense probe (**J**-**L**) showing ZAR1 expression in egg cell and synergids (**J**), central cell (**K**), zygote and endosperm (**L**) and *ZAR1* sense probe (**M**). cc, central cell; cn, central cell nucleus; ec, egg cell; ed, endosperm; en, endosperm nucleus; sc, synergid cell; sn, synergid nucleus; sp, sperm nucleus; vn, vegetative nucleus; zy, zygote. Bars = 10 μm in (**D**-**G**) and (**J**-**M**). Bars = 5 μm in (**H-I**).

Molecular analysis indicates that *zar1-1*^*+/-*^ contains a single *Ds* insertion which causes a 377 bp deletion from +1516 bp in the intron to +1893 bp in the second exon of *At2G01210*. And in the two weak alleles, *zar1-2* and *zar1-3*, which contain a *Ds* insertion and a *T-DNA*, respectively, the extracellular region of At2G01210 is interrupted in both *zar1-2* and *zar1-3* (Fig 4A and S3 Fig).

To investigate whether *At2G01210* is the *ZAR1* gene, we introduced a 3.5 kb genomic DNA fragment spanning the locus into *zar1-1*^*+/-*^ mutant plants. The transgene decreased the seed abortion rate from 26.3% (n = 962) in *zar1-1*^*+/-*^ to 19.8% (n = 951) to 8.4% (n = 965) in different T1 transgenic lines (S4A Fig). Statistical analysis showed the abortion rate of ovules is decreased significantly in transgenic plants compared to that of *zar1-1*^*+/-*^. These demonstrate that the transgene can rescue *zar1-1*^*+/-*^ phenotype. We also introduced this 3.5 kb *ZAR1* genomic fragment into *zar1-2* mutant and checked the phenotype of transgenic plant. It shows that the selected *ZAR1* genomic fragment can rescue the *zar1-2* phenotype and the zygote division in *zar1-2* Is very similar to the wild-type plants. Interestingly, the wild-type plants transformed with *pzar1*:*ZAR1*^*ΔK*^*-GFP*, in which the kinase domain of ZAR1 was replaced by GFP to mimic the *Ds* insertion in *zar1-1*, reduced the seed set from 95.81% (n = 682) to 76.03–93.04% (n > 630) in different transgenic lines. The lowest seed set is close to *zar1-1*^*+/-*^ (73.22%) (n = 785). Quantitative RT-PCR analysis indicates that the mRNA level of the *ZAR1*^*ΔK*^*-GFP* correlates tightly with the seed set reduction (S4B Fig). Moreover, the extra-long zygote or daughter cells and approximately symmetric division of zygote seen in *zar1-1*^*+/-*^ were also observed in *pzar1*: *ZAR1*^*ΔK*^*-GFP* plants (S5 Fig). Together, these data confirmed that the *At2G01210* is indeed the *ZAR1* gene.

The above data also suggest that the phenotypic difference may be resulted from different nature of the alleles. To investigate this possibility, we first checked if the truncated mRNA is produced. Indeed, there is a truncated transcript of about 800 nt in *zar1-1*^*+/-*^ plants besides the 1500 nt wild-type mRNA (S6A and S6B Fig). Furthermore, the presence of a 42 KD truncated protein in *zar1-1*^*+/-*^, but not in *zar1-2*, was confirmed with Western analysis using ZAR1 antibody (S6C Fig). These data indicate that *zar1-2* is a null mutation and the sterile phenotype in *zar1-1*^*+/-*^ is most likely caused by the truncated protein.

ZAR1 is a protein of 716 amino acids with an N-terminal signal peptide, sequentially followed by seven LRR repeats, a transmembrane domain (TM), and a C-terminal serine/threonine kinase domain which consists of eleven subdomains (VI-XI) (Fig 4A and S3 Fig). There is a putative CaM-binding motif (CaMBD) downstream the TM, and a putative Q (D/E) RQQ-type Gβ-binding motif [22] between subdomain VII and VIII. ZAR1 is grouped to the Type III LRR-RLK subfamily [23]. The *Ds* is inserted into the subdomain V in *zar1-1*, and a *Ds* or *T-DNA* is inserted in the region between LRR7 and TM domain in *zar1-2*, or *zar1-3*, respectively (Fig 4A and S3 Fig).

To investigate ZAR1 subcellular localization, CaMV 35S promoter driving *ZAR1-GFP* fusion gene was constructed and introduced into Arabidopsis. Confocal microscopy on transgenic roots showed that ZAR1-GFP fusion protein is co-localized with membrane dye FM4-64 in plasma membrane (Fig 4B and 4C). This indicates that ZAR1 is a plasma membrane receptor-like protein kinase.

To investigate the expression pattern of *ZAR1*, *pZAR1*:*NLS-YFP* fusion was made and introduced into Arabidopsis. We found that *ZAR1* is detected at the micropylar nuclei of the embryo sac at eight-nucleate stage (FG5 stage) before cellularization, and no YFP signal is observed in chalazal nuclei (Fig 4D). After cellularization, the YFP signal is specifically detected in central cell and synergids in mature embryo sac (Fig 4E) with *pZAR1*:*NLS-YFP* transgenic plants. After fertilization, the YFP signal is detected specifically in zygote, endosperm precursor cell, and later in endosperm (Fig 4F and 4G). In addition, YFP signal is also present in sperm and the vegetative cells in both pollen and pollen tube (Fig 4H and 4I). Since it is very difficult in our hands to observe YFP signal in mature egg cell, we conducted RNA *in situ* hybridization. The result indicated that *ZAR1* is expressed at high level in the egg cell (Fig 4J), the central cell (Fig 4K), the endosperm, and at low level in the zygote (Fig 4L), While no signal was detected in the control hybridized with the sense RNA probe (Fig 4M). Together, these indicated that *ZAR1* is specifically expressed in the mature gametophytic cells and the product of double fertilization.

### ZAR1 physically interacts with CaM in Arabidopsis

**Fig 5:**
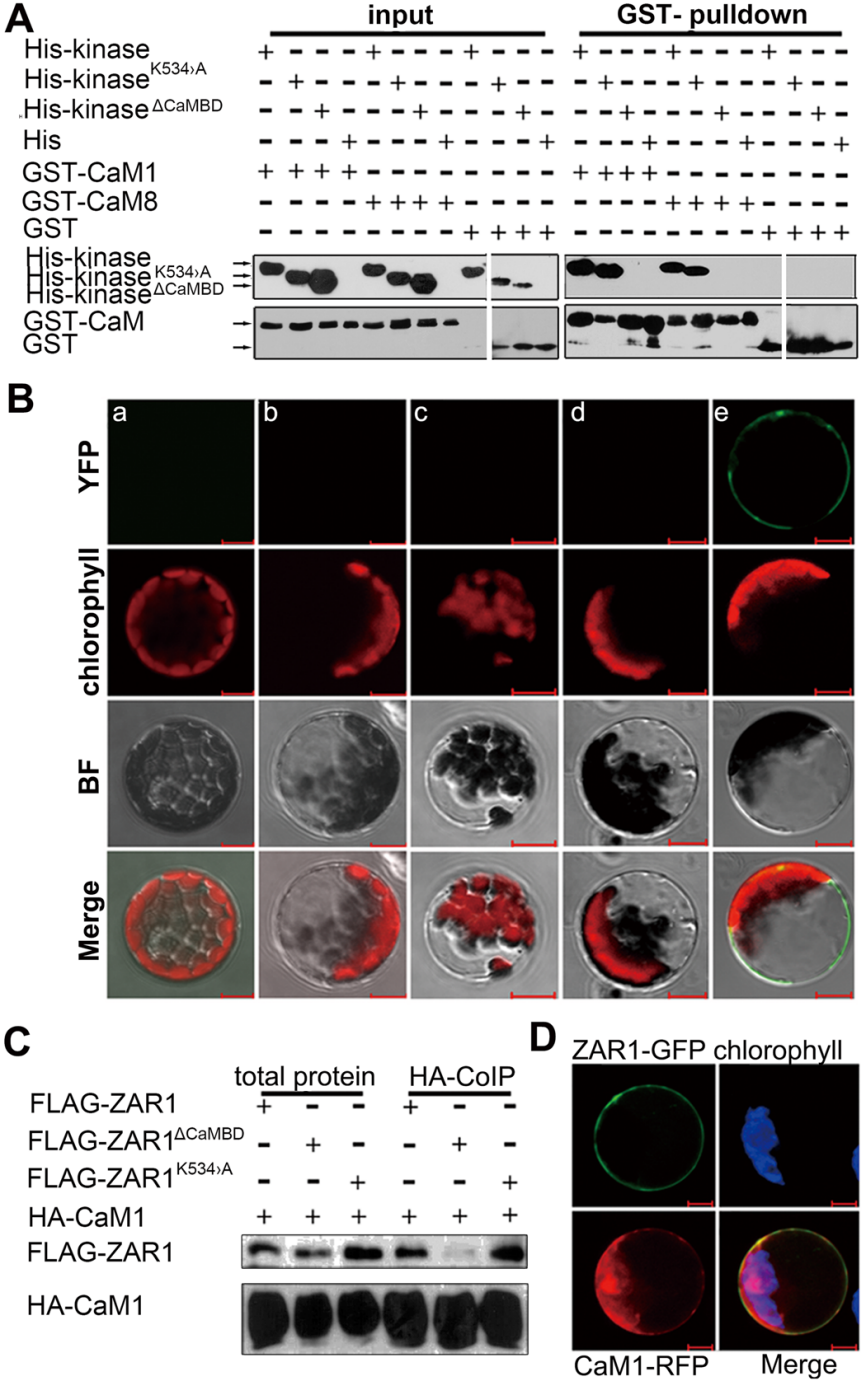
ZAR1 interacts with CaM1. (**A**) Pull-down assay showing interaction between His-kinase with GST-tagged CaM1 or CaM8 in a CaMBD dependent manner. (**B**) BiFC assay in Arabidopsis leaf protoplasts. No YFP reconstitution in control combinations: *pCaM1-nYFP/pcYFP*, *pZAR1-cYFP/pnYFP*, *pZAR1-cYFP/pPMS1-nYFP*, or *pZAR1*^*ΔCaMBD*^*-cYFP/pCaM1-nYFP*. YFP is restored only with *pZAR1-cYFP/pCaM1-nYFP*, indicative of the interaction between ZAR1 and CaM1 in a CaMBD dependent manner. a, *pCaM1-nYFP* and *pcYFP*; b, *pnYFP* and *pZAR1-cYFP*; c, *pPMS1-nYFP* and *pZAR1-cYFP*; d, *pCaM1-nYFP* and *p*Z*AR1*^*ΔCaMBD*^*-cYFP*; e, *pCaM1-nYFP* and *pZAR1-cYFP*. (**C**) Co-IP assays showing ZAR1 and CaM1 interaction. FLAG-tagged ZAR1 and its variants were co-expressed with HA-tagged CaM1 in Arabidopsis leaf cells and FLAG-ZAR1 was co-immunoprecipitated with CaM1-HA using anti-HA antibody. (**D**) Co-localization of ZAR1-GFP (green) with CaM1-RFP (red) in plasma membrane. Bars = 20 μm.

To investigate whether ZAR1 interacts with CaM as suggested by the putative predicated CaM-binding motif. First, we performed protein pull-down experiment with proteins expressed in *Escherichia coli*. Constructs producing His-tagged ZAR1 kinase domain (shorted as “His-kinase” in following text and the figures), His-kinase^K534>A^ with Lys534 to Ala534 mutation, and His-kinase^ΔCaMBD^ (kinase domain with CaMBD deletion), and GST-tagged CaMs, were made and expressed in bacteria. The pull-down results showed that His-kinase and His-kinase^K534>A^, but not His-kinase^ΔCaMBD^, can be pulled down by either GST-CaM1 or GST-CaM8 (Fig 5A and S7 Fig). The interaction between ZAR1 and CaM1 was independent of the concentration of Calcium, but the interaction was enhanced when the concentration of Calcium increased from 2 to 10 μmol/L (S7 Fig). It shows that the ZAR1 kinase domain can physically interact with CaM1 and CaM8 via its putative CaM-binding domain *in vitro*, while the kinase catalytic activity is not essential for the interaction.

To further confirm the above *in vitro* interaction between ZAR1 and CaM1 in plant cells, bimolecular fluorescent complementation (BiFC) and co-immunoprecipitation (Co-IP) assay were carried out. In BiFC experiment, Arabidopsis leaf protoplast was co-transformed with *p35S*:*CaM1-nYFP* paired with different *ZAR1* constructs; meanwhile, *p35S*:*PMS1-nYFP* was introduced as a negative control. YFP fluorescence is only detected in the plasma membrane in cells co-expressing *p35S*:*ZAR1-cYFP* and *p35S*:*CaM1-nYFP* (Fig 5B), indicating that the interaction between ZAR1 and CaM1 occurs at plasma membrane. These interactions were also confirmed by Co-IP assay with proteins from transformed Arabidopsis leaf protoplasts, namely the HA-CaM1 co-immunoprecipitates with either FLAG-ZAR or FLAG-ZAR ^K534>A^, but not with FLAG-ZAR1^ΔCaMBD^ (Fig 5C). This strongly indicates that CaM1 interacts with ZAR1 via the CaMBD domain *in vivo*. Finally, we checked whether ZAR1 and CaM1 are co-localized in cells as CaMs are cytosolic proteins. When we observed Arabidopsis protoplasts transformed with *pZAR1*:*ZAR1-GFP* and *p35S*:*CaM1-RFP*, we found the co-localization of ZAR1-GFP and CaM1-RFP at the plasma membrane, in addition to the localization of ZAR1-GFP at plasma membrane, and most CaM1-RFP is in cytoplasm (Fig 5D). Together, these data showed that ZAR1 interacts with CaM1 via its CaM-binding domain at the plasma membrane *in planta*.

### ZAR1 interacts with Gβ subunit

**Fig 6:**
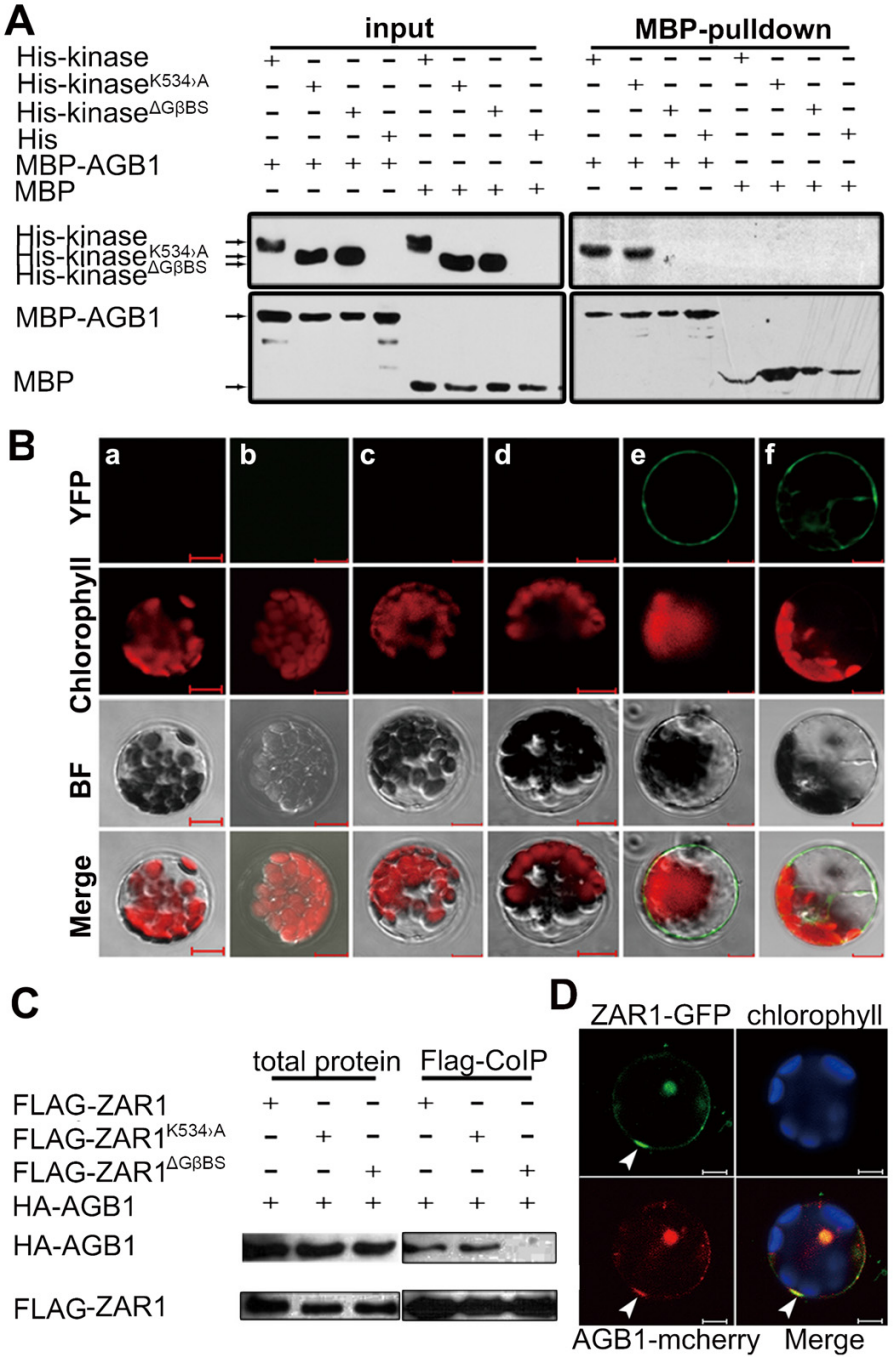
Interaction between ZAR1 and AGB1. (**A**) Pull-down assay showing interaction of His-kinase or its variants with MBP-tagged AGB1 in a GβBS dependent manner. (**B**) BiFC assays in Arabidopsis leaf cells. No YFP restoration in control combinations: *pAGB1-nYFP/pcYFP*, *pZAR1-cYFP/pPMS1-nYFP*, *pZAR1*^*ΔGβBS*^*-cYFP/pAGB1-nYFP*, and *pZAR1-cYFP/pnYFP*. YFP was restored in *pZAR1-cYFP/pAGB1-nYFP* combinations, indicative of ZAR1 and AGB1 interaction. a, *pAGB1-nYFP* and *pcYFP*; b, *pnYFP* and *pZAR1-cYFP*; c, *pPMS1-nYFP* and *pZAR1-cYFP*; d, *pAGB1-nYFP* and *p*Z*AR1*^*ΔGBS*^*-cYFP*; e and f, *pAGB1-nYFP* and *pZAR1-cYFP*. (**C**) Co-IP assay showing interaction of ZAR1 and AGB1 *in vivo*. FLAG-tagged ZAR1 and its variants were co-expressed with HA-tagged AGB1 in Arabidopsis leaf cells. HA-AGB1 was co-immunoprecipitated by FLAG-ZAR1 using anti-FLAG antibody. (**D**) Co-localization of ZAR1-GFP with AGB1-mCherry in plasma membrane foci (arrowheads). Bars = 20 μm.

The presence of a putative glutamine-rich Gβ-binding site (GβBS) in ZAR1 suggests that ZAR1 may interact with Gβ although such interaction has not been demonstrated before. We performed pull-down, BiFC and Co-IP experiments to check the interaction between ZAR1 and Gβ. Both His-kinase and His-kinase^K534>A^ proteins, but not His-kinase^ΔGβBS^ (kinase domain with GβBS deletion), were pulled down with MBP-AGB1 (Fig 6A). Consistently, such interaction was confirmed by BiFC (Fig 6B) and Co-IP experiments (Fig 6C). YFP fluorescence was clearly detected in plasma membrane and also in endomembrane in protoplasts co-expressing ZAR1-cYFP and AGB1-nYFP. Interestingly, the YFP fluorescence is unevenly distributed on the plasma membrane (Fig 6B). HA-AGB1 protein co-precipitated with FLAG-ZAR1 and FLAG-ZAR^K534>A^, but not with FLAG-ZAR1^ΔGβBS^ (Fig 6C). These data showed that ZAR1 interacts with AGB1 via its Gβ-binding motif *in vivo* and *in vitro*.

As shown above, ZAR1 is a plasma membrane protein, and AGB1 is a protein localized on plasma membrane and in nucleus [24], we questioned whether they can meet spatially in cells. Surprisingly, ZAR1-GFP is co-localized with AGB1-mCherry in foci on the plasma membrane and also in nucleus in protoplasts co-transformed with *p35S*:*ZAR1-GFP* and *p35S*:*AGB1-mCherry* (Fig 6D). Taken together, our data show that ZAR1 interacts with AGB1 indeed, and they may function together on the membrane or in the nucleus as well.

**Fig 7:**
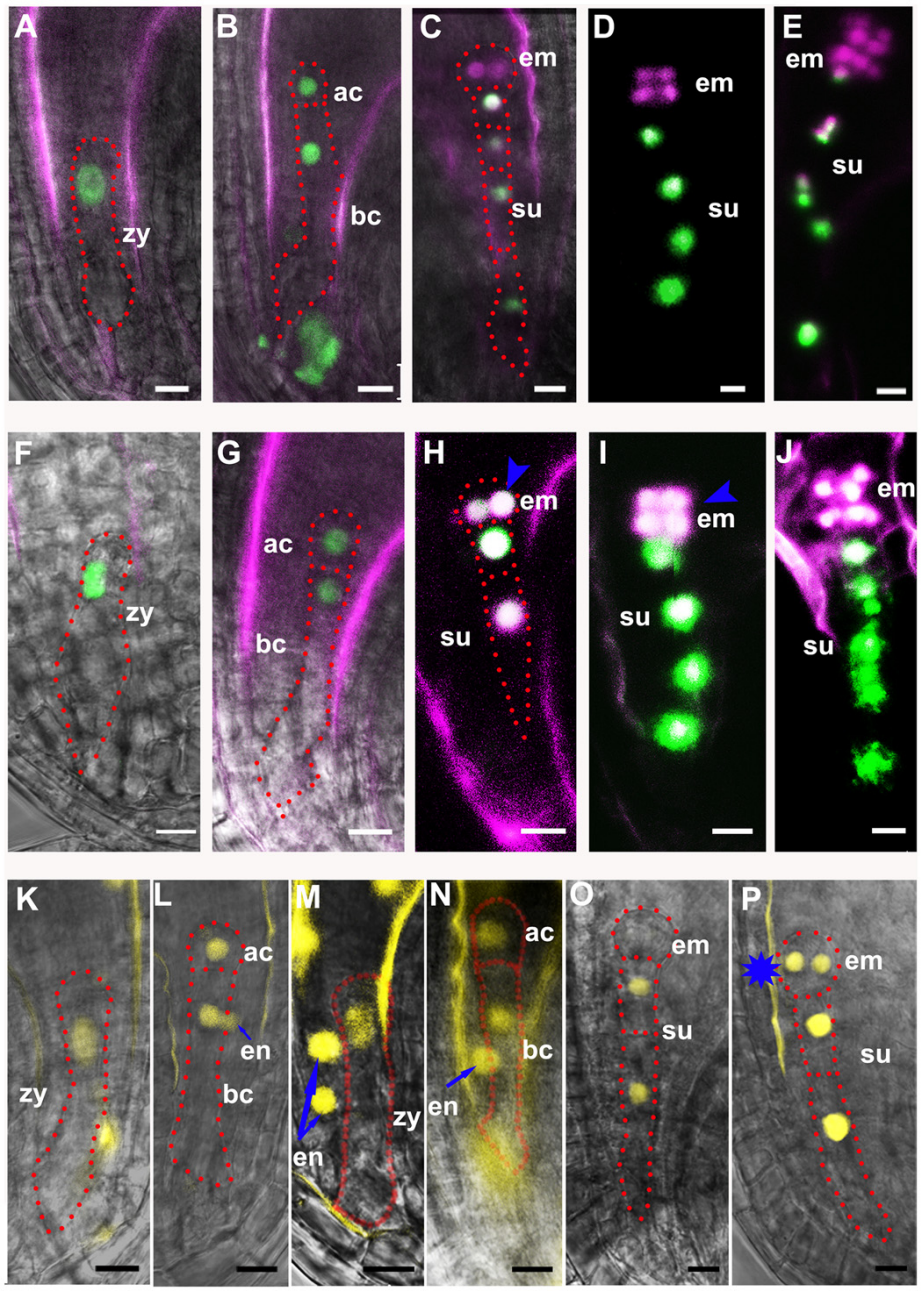
Ectopic expression of cell lineage markers in *agb1-2* mutants. (**A-J**) Basal cell-specific marker *pWOX8gΔ*:*NLS-vYFP*_*3*_ and apical cell specific-marker *pWOX2*:*DsRed2* were expressed in the wild-type (**A-E**) and *agb1-2* proembryos *(***F-J**). The basal marker is mis-expressed in the apical lineage besides the basal cell lineage in *agb1-2* embryo (blue arrowhead) (**H-J**). YFP signal is falsely colored with green and DsRed signal with purple. (**K-N**) Expression of zygote asymmetric division marker *pWRKY2*:*NLS-vYFP*_*3*_ in the wild-type (**K-L**) and *agb1-2* (**M-N**) proembryos. Blue arrows show the ectopic expression of *pWRKY2*: *NLS- vYFP*_*3*_ in endosperm of *agb1-2* proembryos. (**O-P**) Expression of basal cell-specific marker *pWOX9*:*NLS-vYFP*_*3*_ in the wild-type (**O**) and *agb1-2* embryos (**P**). Star shows ectopic expression of basal cell lineage marker *pWOX9*:*NLS-vYFP*_*3*_ in apical cell lineage of *agb1-2*. ac, apical cell; bc, basal cell; em, embryo; en, endosperm; su, suspensor; zy, zygote. Bars = 10 μm.

The interaction between ZAR1 and AGB1 prompted us to check if zygote division is also impaired in *agb1-2*. Similar to *zar1-2*, *abg1-2* is a fertile mutant showing endurable symmetric division of the zygote. We compared the length of apical cell and basal cell which could be measured under microscope. The length of the wild-type apical and basal cells are 16.45±1.84 μm (n > 30), and 58.90±5.12 μm (n > 30) respectively (Fig 2A, 2F and 2G). However, *zar1-1* has longer apical cell (27.27±2.53 μm, n > 30) and basal cells (78.96±4.85 μm, n > 30) (Fig 2B, 2F and 2G), *zar1-2* shows a similar apical cell, but a significantly shortened basal cell (Fig 2C, 2F and 2G), while the *agb1-2* apical cell is slightly shortened (Fig 2D and 2F) but the basal cell is dramatically decreased to 44.84±3.42 μm (n > 30) (Fig 2D and 2G) compared with the wild-type. It suggests that the zygote asymmetric division in *zar1-1*, *zar1-2*, and *agb1-2* ovules is disrupted. Interestingly, in *zar1-2 agb1-2* double mutants, the length of basal cells is 78.55±4.83 μm (n > 30) (Fig 2E and 2G), very approximate to that of *zar1-1* basal cells, which is attributed to the non-interaction between AGB1 and ZAR1^ΔK^ in *zar1-1* (the AGB1-binding site is deleted in the *zar1-1*). It implies that the asymmetric division of zygote and the development of basal cell are impaired because of the mutation of *ZAR1* and *AGB1*. Moreover, expression pattern of cell linage-specific markers in *agb1-2* is very similar to that in *zar1-2* (Fig 3I–3N), for example, the basal cell-specific marker, *pWOX8gΔ*:*NLS-vYFP*_*3*_, is also ectopically expressed in embryo proper in *agb1-2* ovules (Fig 7F–7J). Likewise, expression of zygote and basal cell marker, *pWOX9*:*NLS-vYFP*_*3*_ is extended to the apical cell lineage in *agb1-2* ovules (Fig 7P) compared with the wild-type (Fig 7O), and *pWRKY2*:*NLS-vYFP*_*3*_ is detected unexpectedly in endosperm besides zygote and its daughter cells in *agb1-2* ovules (Fig 7K–7N) too. It suggests that ZAR1 and AGB1 play roles in the asymmetric division of zygote and specification of apical and basal cell lineages, and they possibly function through their interaction in the same, or overlapping pathway during early embryogenesis.

### CaM1 and Gβ form complex with ZAR1 and promote ZAR1 kinase activity

**Fig 8:**
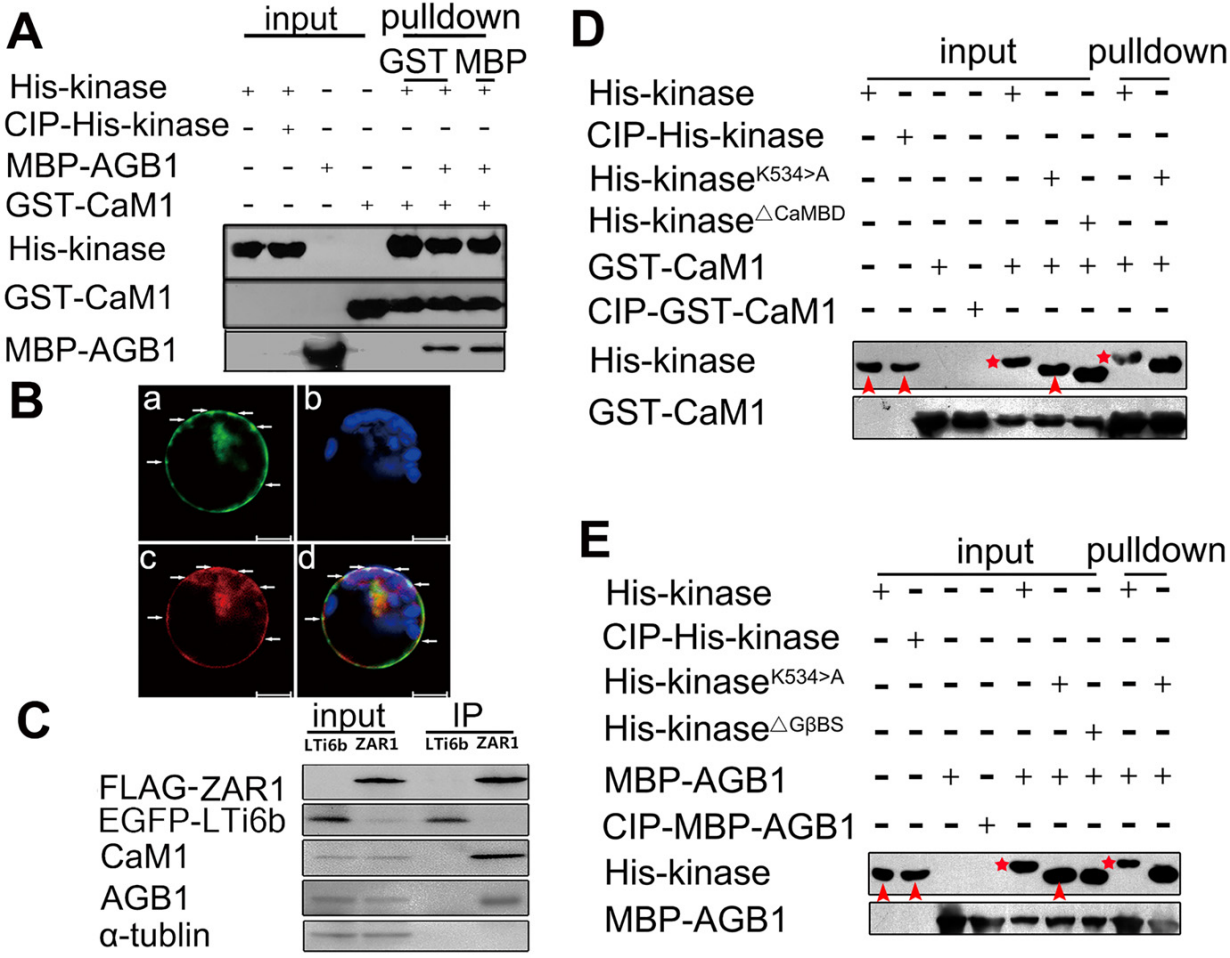
ZAR1’s interaction with CaM1 and AGB1 is activated by the binding. (**A**) Pull-down assay showing that ZAR1 forms complex with CaM1 and AGB1. His-tagged ZAR1 intracellular domain was co-incubated with GST-CaM1 and MBP-AGB1. Either CaM-GST or AGB1-MBP pulled down the other two proteins. (**B**) Co-localization of ZAR1 with CaM1 and AGB1 in leaf cells. YFP fluorescence restored by ZAR1-cYFP and AGB1-nYFP interaction (green) is co-localized with CaM1-RFP (red) in plasma membrane foci. a, *pZAR1-cYFP* and *pAGB1-nYFP*; b, chlorophyll; c, *pCaM1-RFP*; d, merge. Bars = 20 μm (**C**) Co-IP assay showing ZAR1 interaction with CaM1 and AGB1 *in planta*. Co-IP was performed using total protein extract from plants overexpressing *FLAG-ZAR1*. Both AGB1 and CaM1 proteins were co-immunoprecipitated with FLAG-ZAR1 using anti-FLAG antibody. EGFP-LTi6b transgenic plant was used as negative control. (**D**) Pull-down assay showing auto-phosphorylation of ZAR1 kinase domain upon binding of CaM1. ZAR1 kinase and its variants tagged with His, CaM1 tagged with GST, CIP treatment as control. (**E**) Pull-down assay showing auto-phosphorylation of ZAR1 kinase domain upon binding of AGB1. ZAR1 kinase and its variants were tagged with His, and AGB1was tagged with MBP. Stars and arrows in D and E indicate phosphorylated and non-phosphorylated kinases, respectively.

To investigate whether ZAR1, CaM1 and AGB1 form complex since ZAR1 interacts with both CaM1 and AGB1, an *in vitro* pull-down experiment was conducted. As shown in Fig 8A, GST-CaM1 pulls down both His-kinase and MBP-AGB1. Similarly, MBP-AGB1 can also pull down both GST-CaM1 and His-kinase (Fig 8A). This indicates that ZAR1, CaM1 and AGB1 are able to form a complex *in vitro*. To check if ZAR1, CaM1 and AGB1 can form complex *in vivo*, we co-express *pZAR1-cYFP*, *pAGB1-nYFP* and *pCaM1-RFP* in Arabidopsis protoplast. It is expected that the interaction between ZAR1 and AGB1 should restore the YFP that will be co-localized with CaM1-RFP if they form a complex. Indeed, the YFP fluorescence was restored and co-localized with CaM1-RFP on plasma membrane (Fig 8B). These further indicate that ZAR1, AGB1 and CaM1 can meet spatially and form complex in plant cells. Consistently, CaM1 and AGB1 co-precipitates with FLAG-ZAR1 in *FLAG-ZAR1* transgenic plants, but not with LTi6b (as a control) (Fig 8C). These data demonstrate that ZAR1, CaM1 and AGB1 form complex *in planta*.

To further investigate whether the binding of CaM1 and AGB1 affects ZAR1 kinase activity, *in vitro* pull-down experiment was performed. As known, calf intestinal alkaline phosphatase (CIP) catalyzes the removal of phosphate groups from the Ser, Thr and Tyr of phosphorylated protein non-specifically. Lys^534^ is the putative kinase catalytic site in ZAR1 kinase, and it is supposed to be responsible to transfer the phosphate group to Ser, Thr and Tyr of substrate. Indeed, His-kinase^K534>A^ loses the kinase catalytic activity. In the absence of CaM1 or AGB1, there is no difference with the migration rate between CIP phosphatase-treated or untreated His-ZAR1kinase and His-kinase^K534>A^ (Fig 8D and 8E). However, in the presence of CaM1 or AGB1, the His-kinase is activated and moves slower than CIP-treated His-kinase and His-kinase^K534>A^ (Fig 8D and 8E, stars), while CaM1 or AGB1 itself was not phosphorylated (Fig 8D and 8E, arrowheads). It indicates that ZAR1’s auto-phosphorylation is promoted by the binding of CaM1 or AGB1, but the binding is independent on the ZAR1 kinase activity, as CaM1 or AGB1 interacts with His-kinase^K534>A^. These data, together, strongly suggest that ZAR1, AGB1 and CaM1 form complex and the ZAR1 kinase activity is activated by the binding of CaM1 and/or AGB1.

## Discussion

The asymmetric division of the zygote is a conserved feature of embryogenesis important for the establishment of the body plan in animals and plants. Here we showed that ZAR1 plays a role in modulating the asymmetric division of zygote and the cell fate determination of its daughter cells. ZAR1 interacts with CaM and Gβ both *in vitro* and *in vivo*. These data suggest that ZAR1 may act as a membrane receptor kinase to form a complex with CaM1 and AGB1, to activate corresponding signaling cascades that modulate the asymmetric division of the zygote.

### Calmodulin interact with ZAR1 to regulate the zygote development

Calcium signaling has long been implicated to participate in fertilization, zygote activation, and establishment of cell polarity in animals and plants [25–27]. In mammals, Ca^2+^ oscillation in egg triggered by sperm is essential for egg activation and the first cleavage [28]. In plants, the Ca^2+^ signal seen after gamete cytoplasmic fusion in the fertilized egg may also play a role during early embryogenesis [25, 29]. In brown algae, zygote polarity is dependent on local increase of Ca^2+^ concentration in pre-S phase [30], and the first asymmetric division of the zygote is determined by the asymmetric distribution of Ca^2+^ and plasma membrane complexes [31]. Ca^2+^ signals are perceived and decoded by diverse Ca^2+^ sensors, such as CaM, Ca^2+^-dependent protein kinase (CDPK), and Ca^2+^/CaM-dependent protein kinase (CaMK), which play crucial roles in a variety of signaling networks by controlling the activities of a battery of target proteins [32–34]. Here, we demonstrate that ZAR1 interacts with CaM and its kinase activity is activated upon CaM binding. The ratio of zygote symmetric division is 22.6% (n = 138) in *pZAR1*:*ZAR1*^*ΔCaMBD*^ transgenic plants. It indicates that the interaction between ZAR1 and CaM1 is required for the asymmetric division of zygote. It is plausible to speculate that ZAR1 receptor kinase, via its interaction with CaM, is able to respond to Ca^2+^ increase triggered by fertilization in the zygote, and translate the signal into developmental cues during zygotic division in Arabidopsis. Therefore, it would be interesting to measure the [Ca^2+^]_cyt_ changes of the egg during fertilization and to investigate the possible links between the pathway that ZAR1 mediated and the Ca^2+^ signaling cascade during zygote development.

### Heterotrimeric Gβ couples ZAR1 during patterning of the early proembryo

Heterotrimeric G proteins, another key molecular switch in cell signaling, are also involved in asymmetric divisions in animals. G proteins control asymmetric cell division by regulating localization of polarity determinants and spindle orientation [35]. Mutations in Gβγ impair asymmetric division in Drosophila [36] and *C*. *elegans* [37]. In yeasts, Gβγ interacts directly with PAK1 kinase to activate CDC42, a critical regulator of cell polarity [38]. Unlike in animals, there are only one canonical α, one β and two γ subunits in Arabidopsis [39], and the GEF is lost in plants during evolution [40]. In plants, heterotrimeric G proteins are mainly involved in immunity, stress response and nitrogen utilization [41–44], their roles in development are interesting. On one hand, they seem dispensable for development since Arabidopsis plants lacking Gα or Gβ develop normally and grow well. On the other hand, they play critical roles in stress response and hormone regulation pathway. For example, Gα/COMPACT PLANT2 (CT2) interacts with CLV2 to control the shoot size in maize [45]; Gβ is involved in root cell proliferation [46, 47] and the plant-specific non-canonical Gγ (AGG3) is required for organ size control in Arabidopsis [48]. In rice, Gα (D1/RGA1) is involved in rice growth by modulating gibberellic acid and brassinosteroid hormone signaling [49, 50]; COLD1 is a regulator of G protein signaling, it interacts with Gα to activate the Ca^2+^ channel for temperature sensing [44]. According to our data, the asymmetric division of zygote and specification of its daughter cells are impaired because of mutations of *ZAR1* and *AGB1*. It is suggested that the disruption of interaction between ZAR1 and AGB1 results in the abnormality of zygote division and the compensatory growth of basal cell. Consistently, the expression pattern of *WOX8* and *WRKY2* is impaired similarly in both *zar1-2* and *agb1-2* mutants. All these suggest that G proteins might function as modulators in zygote division and the determinants for cell fate of apical and basal cells. Moreover, when we transformed *pZAR1*:*ZAR1*^*ΔGβBS*^ into the wild-type, the symmetric division of zygote was found in T1 generation (15.8%, n = 123). These support the idea that ZAR1 and AGB1 act together to control the asymmetric division of zygote and cell fate determination of its daughter cells.

As discussed above, ZAR1 interacts directly with both CaM and Gβ, and as a membrane receptor kinase, ZAR1 is able to integrate extracellular signal mediating the intracellular Ca^2+^ and heterotrimeric G protein pathways that control zygotic division. There are many details remain to be elucidated during this fundamental process, for example, putative ligand for ZAR1 needs to be identified. Recent studies show that secreted cysteine-rich peptides act as ligand for several LRR-RLKs to regulate asymmetric division during stomata development and cell-cell signaling in fertilization and zygote development [51–57]. It would be worthy to investigate whether such peptides are also ligands for ZAR1 during fertilization.

### The truncated ZAR1 functions as dominant negative mechanism in *zar1-1*^*+/-*^

Our data also indicate that ZAR1 plays a redundant role in controlling zygotic cell division. A stronger phenotype is seen in *zar1-1*^*+/-*^ than in *zar1-2* null allele although asymmetric division of the zygote is disrupted in both *zar1-1*^*+/-*^ and *zar1-2* plants. The strong phenotype in *zar1-1*^*+/-*^ is most likely caused by the truncated ZAR1 protein. Moreover, in *zar1-1*^*+/-*^, the truncated ZAR1 protein likely functions in a dominant-negative and dosage-dependent manner. Indeed, the phenotype depends largely on the expression level of the *pZAR1*:*ZAR1*^*ΔK*^*-GFP* transgene (S4B Fig). On the other hand, the null allele *zar1-2* and *zar1-3* plants are completely fertile although they show a weak zygotic division phenotype. Although both *zar1-1* and *zar1-2* affect zygote development, their phenotypes differ. This raises the question of how the mutations in the *zar1-1* and *zar1-2* alleles affect the wild type molecular function of *ZAR1*. Currently, this cannot be solved easily, more *in vivo* studies on ZAR1 protein and its interacting partners may provide clues.

Dominant negative mutations are common for LRR-RLKs and invaluable for deciphering their functions in plants. For example, all the intermediate and strong alleles of *clavata1* contain missense mutations in the LRR domain and are dominant-negative, the null alleles show very weak phenotype [58]. Another example is the tomato protein Cf-9. Dominant-negative interference by truncated Cf-9 proteins results in leaf chlorosis accompanying leaf necrosis from the base towards the apex in tomato [59, 60]. Similarly, the disease resistant LRR-RLK Xa-21 is capable of functioning without a kinase domain [61]. In *ERECTA*:*ERECTA*^*ΔKinase*^ transgenic plants, the endogenous ERECTA signal is interrupted by highly stable truncated protein by dominant-negative mechanism in a dosage-dependent manner [62]. Our data show that ZAR1 and ZAR1Δkinase co-exist in *zar1-1* (S6C Fig). These imply that the strong phenotype in *zar1-1*^*+/-*^ plants is most likely caused by interference of ZAR1Δkinase with endogenous ZAR1 or ZAR1-interacting partners.

The weak, endurable zygotic block seen in *zar1-2* homozygous plants suggests that additional proteins may take over ZAR1 function when ZAR1 is absent. Such functional redundancy is common for LRR-RLKs since the RLK/Pelle kinase family have been greatly expanded to 374–2205 members in flowering plants, of which the LRR-RLKs represent the largest subfamily with over 223 members in Arabidopsis [23, 63]. Interestingly, there are six LRR-RLK genes that show alternative splicing for proteins of different version, including some truncated proteins [23]. This suggests that the truncated products may function as intrinsic regulators of the RLK signaling system in plants. It has been proposed that multiple receptors may act in a functionally related manner [58, 63, 64]. It is possible that ZAR1 is functionally redundant with related RLKs since null alleles are fertile. Therefore, it would be interesting to identify the ligand and the receptor kinases that functionally overlapped with ZAR1 in regulating the asymmetric division of zygote in Arabidopsis.

### ZAR1 and heterotrimeric G protein regulate the cell fate of zygote daughter cells

Our data propose that ZAR1 plays role in the cell fate specification of basal cell and apical cell. Asymmetric cell divisions generate cells with different fate. In most angiosperms, such as Arabidopsis, zygote undergoes asymmetric division and produces a small apical cell and a large basal cell [20, 21]. The apical and basal cell lineages have different cell fate manifested by differential expression of cell-specific markers [15, 17]. In *zar1-1*^*+/-*^ and *zar1-2* mutants, the elongation of the zygote is very similar to that of the wild-type, but the first asymmetric division is either abolished or transited into an approximately symmetric division, configuring with the shortened basal cell. Interestingly, the basal cell-specific marker *pWOX8gΔ*:*NLS-vYFP*_*3*_ is not expressed in the elongated or symmetrically divided zygote in *zar1-1*^*+/-*^ mutant. While in young proembryos that by-passed the arrest in *zar1-2* and *agb1-2*, *pWOX8gΔ*:*NLS-vYFP*_*3*_ is expressed in both the basal and apical cell lineages, indicating the mis-specification of cell fate, and the cell specification of basal cell and apical cell is disrupted. Both in *zar1-2* and *agb1-2*, *pWRKY2*:*NLS-vYFP*_*3*_ is much more accumulated in endosperm. These suggest that ZAR1 plays a role in defining the expression pattern of *WOX8* and *WRKY2*, and so far cell fate differentiation.

There are over 220 LRR-RLK genes in Arabidopsis genome, 20 LRR-RLKs contain a putative CaM-binding domain and 19 contain a putative Gβ-binding motif. 15 LRR-RLKs have been experimentally proved to interact with CaM, but none of the 19 LRR-RLKs have been shown to interact with Gβ although AT5G67280 has been implicated to interact with Gβ protein genetically [42]. Our data show that ZAR1 interacts with Gβ subunit of heterotrimeric G protein, and support the bold idea that the LRR-RLKs of plants fulfill equivalent roles to GPCRs in fungi and animals in cell-cell signaling [42, 45].

Taken together, we identified a membrane receptor kinase ZAR1 that is required for zygote asymmetric division and the cell fate of its daughter cells. Through its interaction with CaM and Gβ, ZAR1 plays a key role in integrating the intracellular Ca^2+^ and heterotrimeric G protein signaling with extracellular cues during early embryogenesis in Arabidopsis. Preliminarily, we found some clues that ZAR1 might participate in SSP/YODA MAPK kinase pathway (S8 Fig). It is well worthy to investigate the link between ZAR1/AGB1 pathway and YODA MAPK kinase cascade signaling.

## Materials and Methods

### Plant material and phenotype analysis

Plants were grown in greenhouse at 22°C with 50–70% humidity and under the light cycle of 16 hrs daylight/8 hrs darkness. *zar1-1*^*+/-*^ and *zar1-2* are *Ds* gene trap lines generated in *Arabidopsis thaliana* ecotype Landsberg *erecta* [19]. *zar1-3* (SALK_021338) was obtained from ABRC in ecotype *A*. *thaliana* Col-0. *agb1-2* [65] was a gift from Dr. Jirong Huang at Shanghai Institute of Plant Physiology and Ecology, CAS. The seeds of EGFP-LTi6b transgenic plant [66, 67] were from Prof. Yurong Bi at Lanzhou University.

### Phenotype analysis

Plant phenotype was performed as described previously [67]. To observe the embryo phenotype, seeds from *zar1-1*^*+/-*^ siliques were mounted in Herr’s solution before observation with a Zeiss Axioskop [67]. Reciprocal crosses between the wild-type and *zar1-1*^*+/-*^ plants were performed as reported before [68].

### RNA extraction and RT-PCR

Total RNA was extracted with TRIzol reagent (Invitrogen) from different tissues of wild-type plants according to the manufacturer’s instruction. The single-stranded *cDNA* was transcribed by superscriptIII (Invitrogen). Real time PCR was carried out using primers listed in S1 Table, and *ACTIN2/8* was used as internal control.

### Molecular analysis and cloning

To obtain the flanking sequence of *Ds* insert, TAIL-PCR was performed as previous report [69] and primers used are listed in S1 Table. For construction of plasmids *pZAR*:*ZAR1* and *pZAR1*:*ZAR1*^*Δk*^*-GFP-nos*, the corresponding fragments were amplified and subcloned in *pCAMBIA1300* (Clontech) or *pCAMBIA2300* (Clontech) with primers listed in S1 Table. Plasmids *p35S*:*ZAR1-GFP* and *p35S*:*ZAR1-FLAG* were provided by Dr. Jia Li [23]. Plasmids *pZAR1*:*ZAR1-GFP*, *p35S*:*CaM1-RFP* and *p35S*:*AGB1-mCherry* which were used for co-localization in protoplast were constructed in *pBlue-SK* (Stratagene) and *pWEN57-RFP* with primers shown in S1 Table as described above. Constructs of *p35S*:*ZAR1-cYFP*, *p35S*:*CaM1-nYFP* and *p35S*:*AGB1-nYFP* for BiFC, were performed as described previously [67]. The variants of point mutations were introduced into *p35S*:*ZAR1-cYFP* by site-directed mutagenesis. *PMS1 (AT4G02460)* encoding a nucleus protein was cloned to construct as a negative control in BiFC assays. For pull-down experiments, the fragment of *ZAR1-kinase* was amplified with primers listed in S1 Table and cloned into *pET28a* (+). The *ZAR1-kinase* mutations were introduced into the *ZAR1kinase* by site-directed mutagenesis. Similarly, the fragments of *AGB1* was amplified and cloned into *pMAL-C-2X*; the full length of *CaM1* and *CaM8* were amplified and cloned into *pGEX-4T-2*. For Co-IP experiments, full length of *ZAR1* was amplified with primers listed in S1 Table, and cloned into *pUC19-35S*:*3FLAG*, the variants of *ZAR1* were generated by site-directed mutagenesis. The fragments of *CaM1* and *AGB1* were amplified and cloned into *pUC19-35S*:*HA* as above [70].

### Subcellular localization and BiFC assays

Protoplasts from Arabidopsis leaves were prepared and transfected with 10 μg purified DNA as described previously [70]. DNA was extracted with CsCl gradient centrifugation or with EndoFree Plasmid Maxi Kit (QIAGEN). Experiments for co-localization of GFP and RFP or mCherry were performed and repeated at least three times. BiFC assays were performed and repeated according to Walter [71]. FM4-64 staining was performed as described previously [72, 73]. Arabidopsis protoplast and root cells were stained with 10 μmol/L FM4-64 and image was completed within 10 min of staining. Images were acquired with confocal LSM510 or LSM780 (Zeiss). Fluorescent signals were detected with an Argon 2 laser for GFP, YFP (excitation, 488 nm or 514 nm; emission, BP500-530 nm or BP500-560 nm emission filter), and chloroplast auto-fluorescence and excitation, 488nm; emission, LP 615 filter), and with He-Ne Laser (excitation, 543 nm; emission BP560-600 or LP600 emission filter) for RFP and mCherry, and He-Ne Laser (excitation, 561nm; emission, BP570-630 emission filter) for FM4-64.

### Protein analysis

The pull-down assay was carried out according to Xiang with slight modification [70]. Overnight culture of *E*. *coli* with different constructs was transferred to fresh medium with 1/50 dilution, then continued with 3 hrs incubation on shaker (250 rpm, 37°C). When the OD600 reaches 0.6, the culture was moved to 22°C, 250 rpm for 30 min, and then IPTG was added to the final concentration of 0.5 mmol/L. The induction was kept for another 6 hrs at 22°C, 250 rpm. The bacterium cells were collected and resuspended with 10 ml Tris buffer (25 mmol/L Tris pH 7.5, 50 mmol/L NaCl, 3 mmol/L DTT, 1 mmol/L PMSF, protease inhibitor Cocktails), and were debrised by ultrasonic. The samples were spun at 4°C, 13,000 rpm for 30 min. The supernatant of 100 μl was kept for input, and 50 μl of the rest supernatant was incubated with glutathione agarose (GE Healthcare) (GST-tagged beads), in 360° shaker at 4°C for overnight. The beads were spun at 500 g for 2 min, and washed for five times with Tris buffer (25 mmol/L Tris pH 7.5, 50 mmol/L NaCl, 3 mmol/L DTT, 1 mmol/L PMSF, protease inhibitors Cocktails, 1% Triton X-100, 0.1% SDS). SDS loading buffer was added before Western detection.

For protoplast Co-IP assay, the leaf protoplasts were co-transfected with 10 μg plasmid DNA of each construct, *pAGB1-HA/pZAR1-FLAG*, *pAGB1-HA/pZAR1*^*K534>A*^*-FLAG*, and *pAGB1-HA/pZAR1*^*ΔGβBS*^*-FLAG*, with aid of PEG/Calcium, and then incubated at 22°C, 45 rpm, for overnight. The protoplast protein was extracted as described previously [71, 72] with native extraction buffer (20 mmol/L HEPES, pH 7.5, 40 mmol/L KCl, 250 mmol/L glucose, 5 mmol/L MgCl_2_, 1mmol/L PMSF, protease inhibitors Cocktails). The protoplast were harvested by soft spin at 100 g, then resuspended with 500 μl native extraction buffer, and vortexed for 30 s to mix. The samples were spun at 6,000 g for 15 min at 4°C, then supernatants were collected and the pellet were resuspended with 100 μl native extract buffer. The samples were subsequently ultrasonicated for 10 second, and Triton X-100 was added to the final concentration 1%. The samples were spun at 100,000 g for 10 min at 4°C, and the supernatants were collected, and diluted with native extraction buffer [70–72]. 50 μl supernatant was reserved as control for total protein. The rest was mixed with prepared anti-FLAG-M2 gels (Sigma) by rotating at 4°C for 1 hr. The samples were spun down at 1000 rpm for 3 min and then washed five times with washing buffer (20 mmol/L HEPES, pH 7.5, 40 mmol/L KCl, 250 mmol/L glucose, 5 mmol/L MgCl_2_, 0.1% Triton X-100). SDS loading buffer was added to samples before detection by Western blot [68].

Co-IP assay was performed with FLAG-ZAR1, and EGFP-LTi6b transgenic plants (used as control for membrane protein) [65]. The inflorescences and siliques 1–2 DAP, or seedlings of 10 DAG (day after germination) from transgenic plants were collected, and Co-IP assays were carried out as described previously [72, 73]. Anti-FLAG-M2 gel was incubated with samples for 30 min, and AGB1 and CaM1 proteins were checked with Anti-AGB1 antibody (Sigma) and anti-CaM antibody (Sigma), respectively.

### RNA *in situ* hybridization

Sample preparation and sectioning for *in situ* hybridization were according to previous reports [74, 75] with minor modification. The plants were emasculated or pollinated manually, and ovules 24 hrs after emasculation or 12–24 hrs after pollination were collected and fixed before sectioning. The *ZAR1* fragment spans the nucleotide sequence from +259 nt to +1160 nt was cloned into pT-GEM, and the linearized plasmid was transcribed *in vitro* by T7 RNA polymerase or SP6 RNA polymerase (Roche) for antisense or sense probes.

### Accession numbers

Sequence data from this article can be found in the GenBank/EMBL or Arabidopsis Genome Initiative database under the following accession numbers: *ZAR1* (*AT2G01210*), *CaM1* (*AT5G37780*), *CaM8* (*AT4G14640*), *AGB1* (*AT4G34460*), *PMS1 (AT4G02460)*.

## Supporting Information

S1 Fig Endosperm development is arrested in *zar1-1* seeds. Endosperm-specific marker *pDD36*:*GFP* expressed in the wild-type (**A, C**) and *zar1-1* arrested seeds (**B, D**) 1 DAP (**A, B**) and 2 DAP (**C, D**) respectively. a, apical cell; b, basal cell, en, endosperm; Zy, zygote. Bars = 10 μm.
(TIF)

S2 Fig WOX2/8 marker expression in *zar1* mutant. The *trans*-heterozygote with *pWOX2*:*DsRed2*/*pWOX8gΔ*:*NLS-vYFP*_*3*_ markers (from *zar1-1*^*+/-*^/*pWOX2*:*DsRed2*/*pWOX8gΔ*:*NLS-vYFP*_*3*_ x *zar1-2*^-/-^/ *pWOX2*:*DsRed2*/*pWOX8gΔ*:*NLS-vYFP*_*3*_) (**A-D**). In about half seeds (n = 142), the expression pattern of *WOX2* and *WOX8* is consistent with the signal in *zar1-1*, which showed a strong expression of WOX2, but weak expression of WOX8 in *trans*-heterozygote (**A-D**). (**E-H**) The split images showing different fluorescence in the wild-type zygote from Fig 3A. (**I-L**) The split images presenting the different fluorescence of *zar1-1* from Fig 3G. YFP signal is falsely colored with green and DsRed signal with purple. Bars = 5 μm.
(TIF)

S3 Fig Sequence and structure of ZAR1 protein. Amino acid sequence of ZAR1 showing its domains and inserts (arrowhead) of different alleles. The conserved amino acids were shown with stars.
(TIF)

S4 Fig *ZAR1* complementation and sterile phenotype of *ZAR1*^*Δk*^ over-expression plants. (**A**) The seed set abortion of *zar1-1*^*+/-*^ is rescued by introduction of *pZAR1*:*ZAR1g*. (**B**) The sterile phenotype of plants transformed with *pZAR1*:*ZAR1*^*Δk*^*-GFP* is correlated with the mRNA level of *ZAR1*^*Δk*^*-GFP*.
(TIF)

S5 Fig The *pZAR1*:*ZAR1*^*ΔK*^*-GFP* transgene mimics the phenotype of *zar1-1*. Compared to the wild type (**A**), the basal cell is elongated in *pZAR1*:*ZAR1*^*ΔK*^ *-GFP* transgenic plants (**B**) and *zar1-1* mutants (**C**). ac, apical cell; bc, basal cell. Bars = 10 μm.
(TIF)

S6 Fig A truncated protein is produced in *zar1-1*. (**A**) *ZAR1* gene structure with insertion sites indicated. (**B**) RT-PCR analysis showing a truncated mRNA in *zar1-1*^*+/-*^. (**C**) Western blot showing the truncated ZAR1 protein (arrowhead) in *zar1-1*^*+/-*^. TUB6 was used as loading control.
(TIF)

S7 Fig The interaction between ZAR1 and calmodulin is independent of the concentration of calcium. The interaction of ZAR1 and CaM1 is shown in pull-down assay (**A**). The statistical analysis indicates that the interaction is enhanced with the increasing concentration of calcium (**B**).
(TIF)

S8 Fig *ZAR1* functions upstream of *SSP* during zygote development. The symmetric division in *zar1-1* (**B**), *ssp* (**C**) and *zar1-1/ssp* (**D**), compared to the wild type (**A**). The immature seeds of *ssp*^*-/-*^/*zar1-1*^+/-^ plants (**D**) showed very similar phenotype to *ssp*^*-/-*^ mutant (**C**). ac, apical cell; bc, basal cell. Bars = 10 μm. (**E**) Pull-down assay showing interaction between His-tagged ZAR1 kinase domain with GST-tagged SSP protein.
(TIF)

S1 Table List of primers. (DOCX)

S2 Table Expression analysis of *pWOX8gΔ*:*NLS-vYFP*_*3*_ in *zar1-2* and *agb1-2*. (DOCX)

S1 Text Supporting materials and methods. (DOCX)
