# Supplementary figures and images for: The Arabidopsis Receptor Kinase ZAR1 Is Required for Zygote Asymmetric Division and Its Daughter Cell Fate
Source: PLoS Genet. 2016 Mar 25;12(3):e1005933. doi: 10.1371/journal.pgen.1005933 (PMC4807781; doi:10.1371/journal.pgen.1005933)

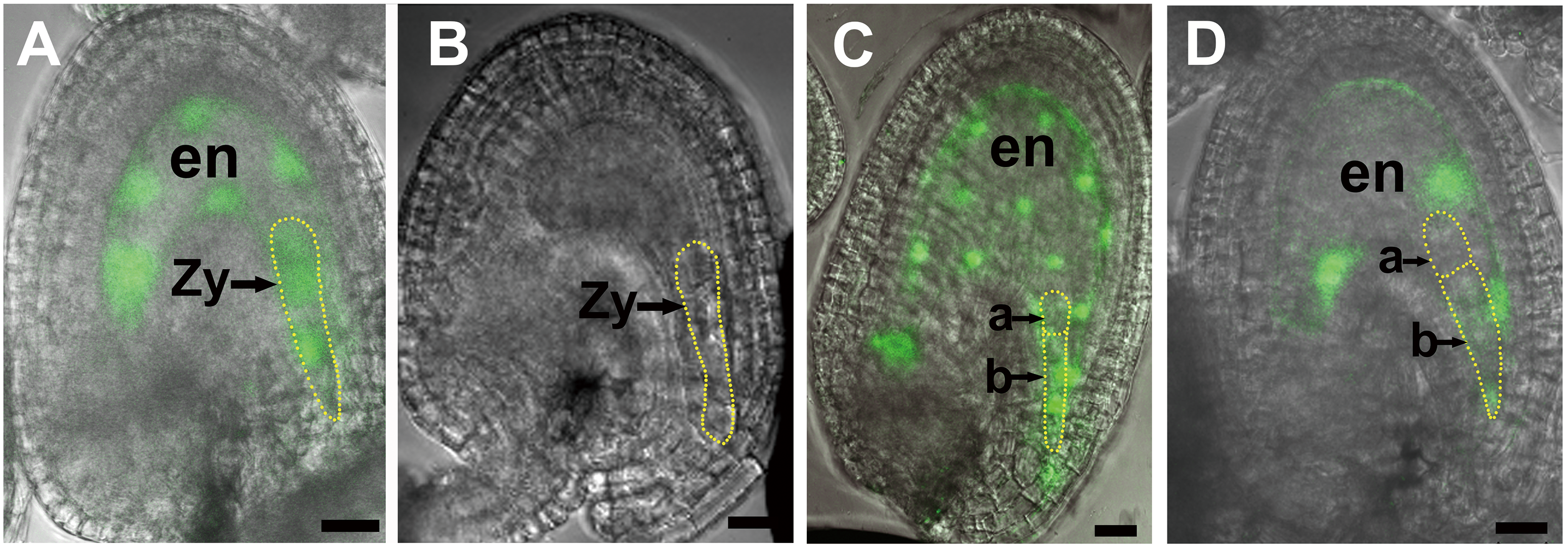

Supplement: S1 Fig — Endosperm-specific marker pDD36:GFP expressed in the wild-type (A, C) and zar1-1 arrested seeds (B, D) 1 DAP (A, B) and 2 DAP (C, D) respectively. a, apical cell; b, basal cell, en, endosperm; Zy, zygote. Bars = 10 μm. (TIF) [file pgen.1005933.s001.tif]

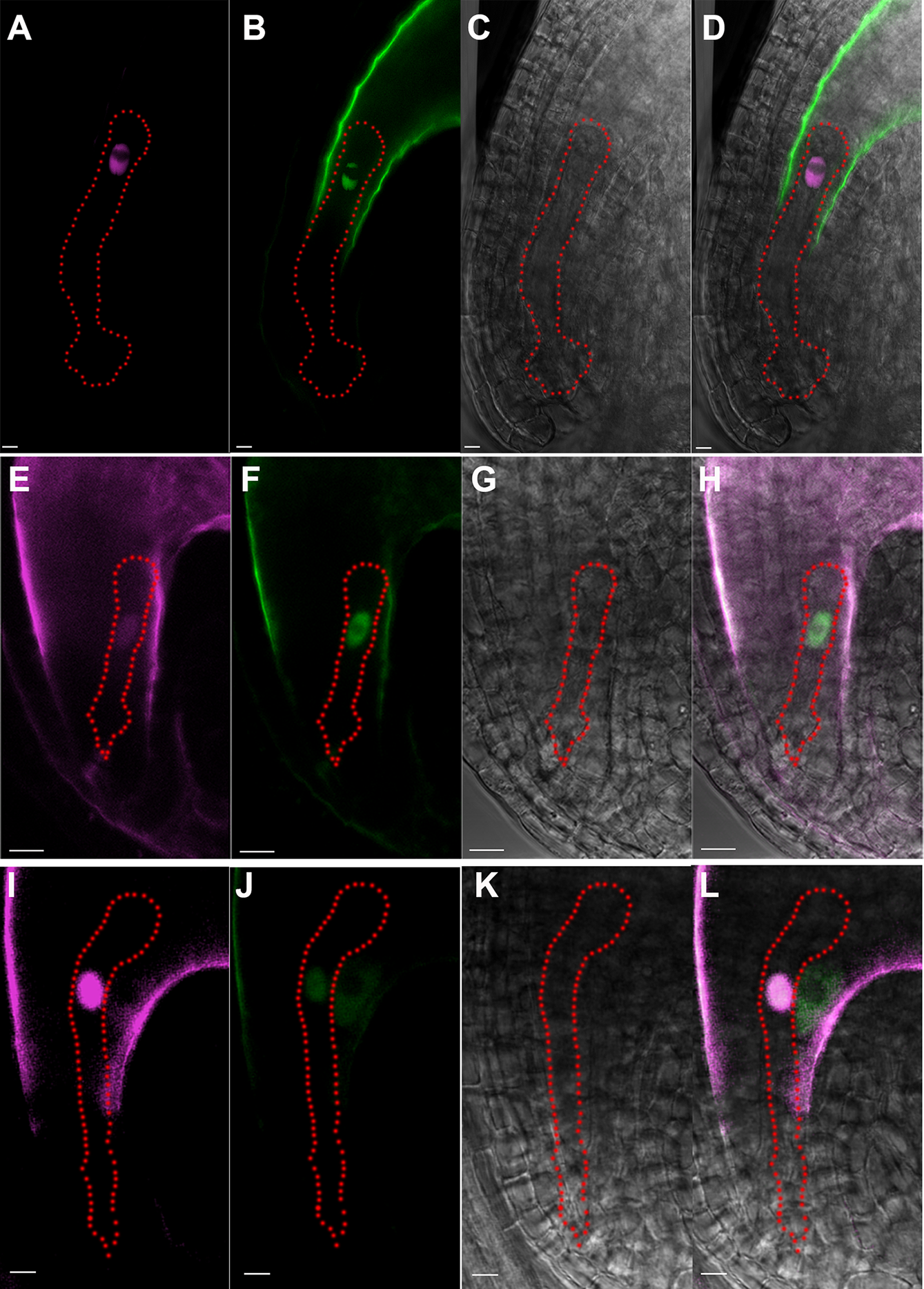

Supplement: S2 Fig — The trans-heterozygote with pWOX2:DsRed2/pWOX8gΔ:NLS-vYFP3 markers (from zar1-1+/-/pWOX2:DsRed2/pWOX8gΔ:NLS-vYFP3 x zar1-2-/-/ pWOX2:DsRed2/pWOX8gΔ:NLS-vYFP3) (A-D). In about half seeds (n = 142), the expression pattern of WOX2 and WOX8 is consistent with the signal in zar1-1, which showed a strong expression of WOX2, but weak expression of WOX8 in trans-heterozygote (A-D). (E-H) The split images showing different fluorescence in the wild-type zygote from Fig 3A. (I-L) The split images presenting the different fluorescence of zar1-1 from Fig 3G. YFP signal is falsely colored with green and DsRed signal with purple. Bars = 5 μm. (TIF) [file pgen.1005933.s002.tif]

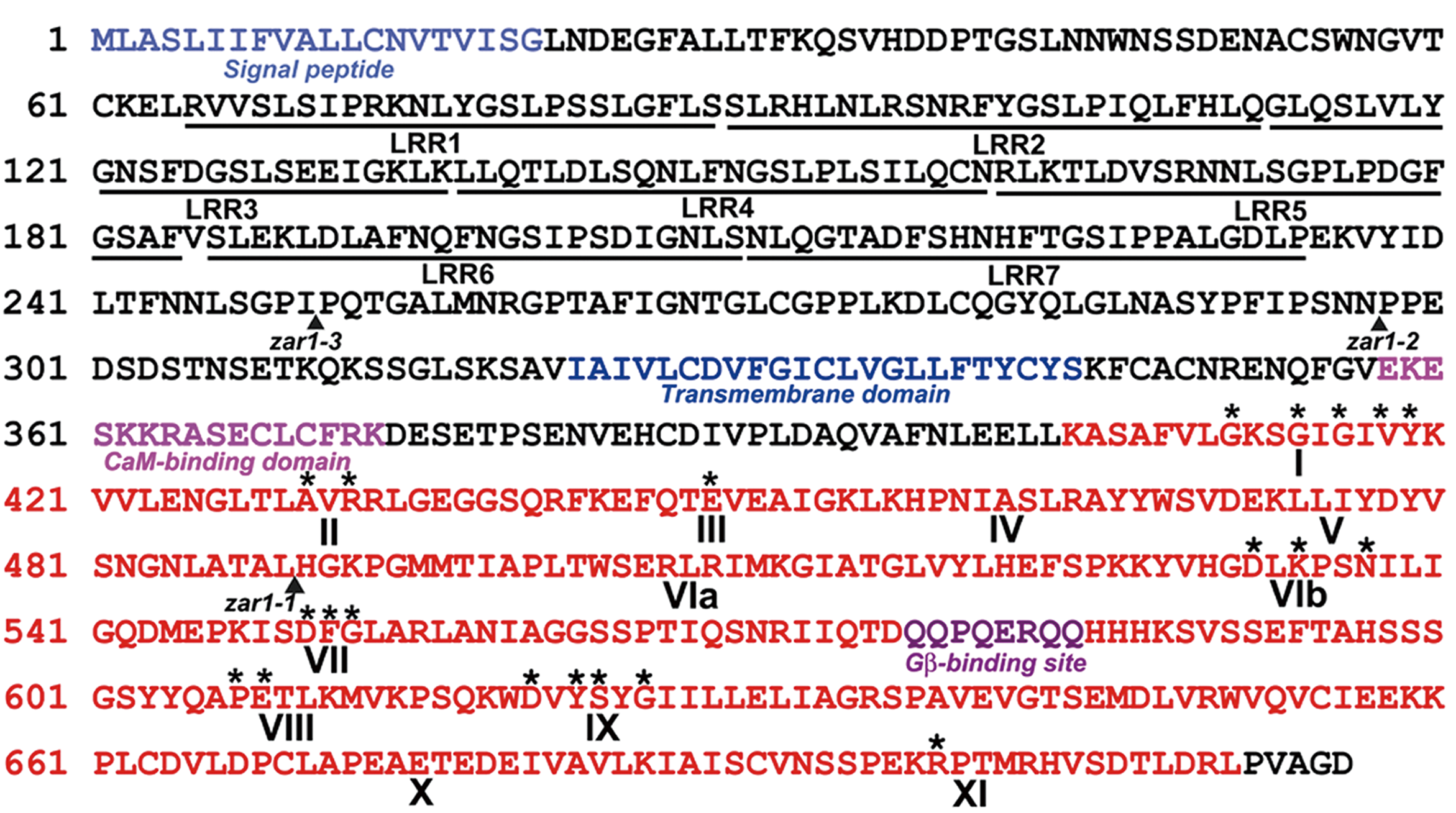

Supplement: S3 Fig — Amino acid sequence of ZAR1 showing its domains and inserts (arrowhead) of different alleles. The conserved amino acids were shown with stars. (TIF) [file pgen.1005933.s003.tif]

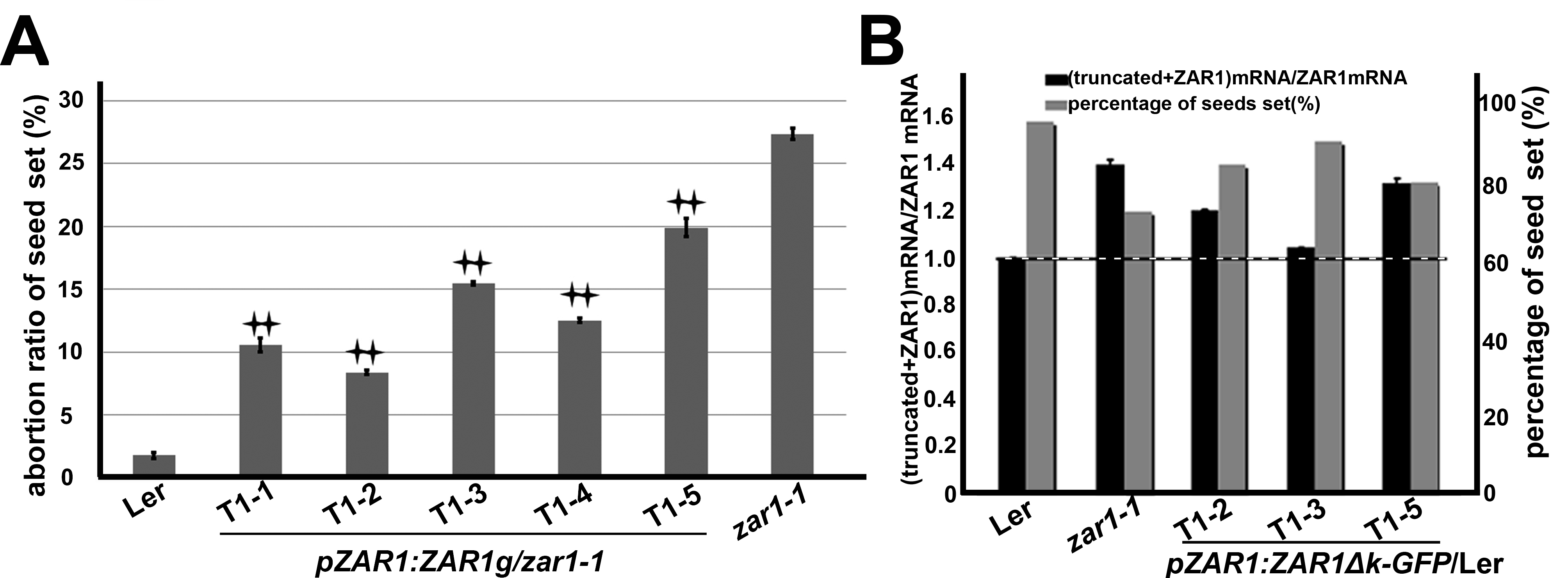

Supplement: S4 Fig — (A) The seed set abortion of zar1-1+/- is rescued by introduction of pZAR1:ZAR1g. (B) The sterile phenotype of plants transformed with pZAR1:ZAR1Δk-GFP is correlated with the mRNA level of ZAR1Δk-GFP. (TIF) [file pgen.1005933.s004.tif]

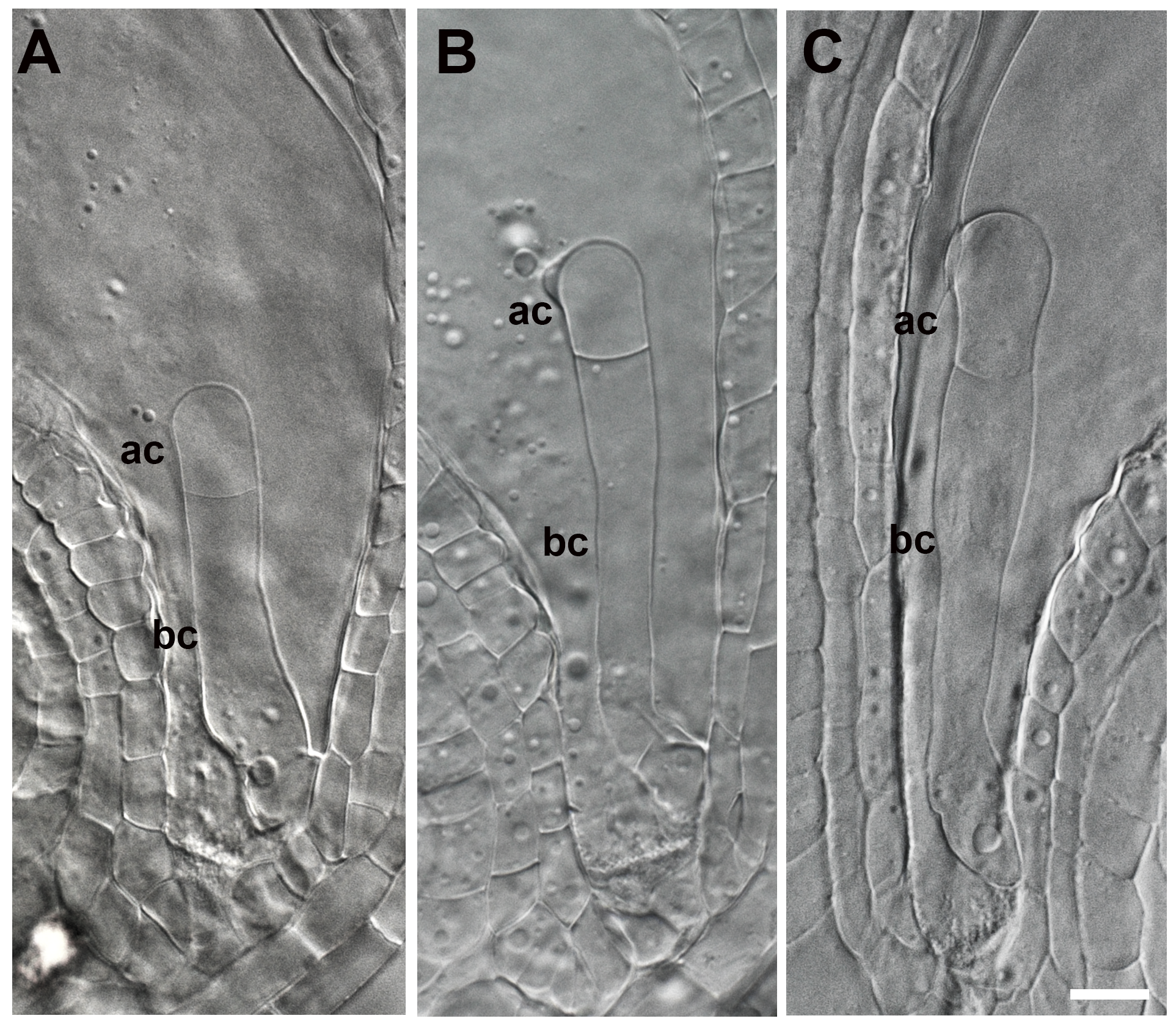

Supplement: S5 Fig — Compared to the wild type (A), the basal cell is elongated in pZAR1:ZAR1ΔK -GFP transgenic plants (B) and zar1-1 mutants (C). ac, apical cell; bc, basal cell. Bars = 10 μm. (TIF) [file pgen.1005933.s005.tif]

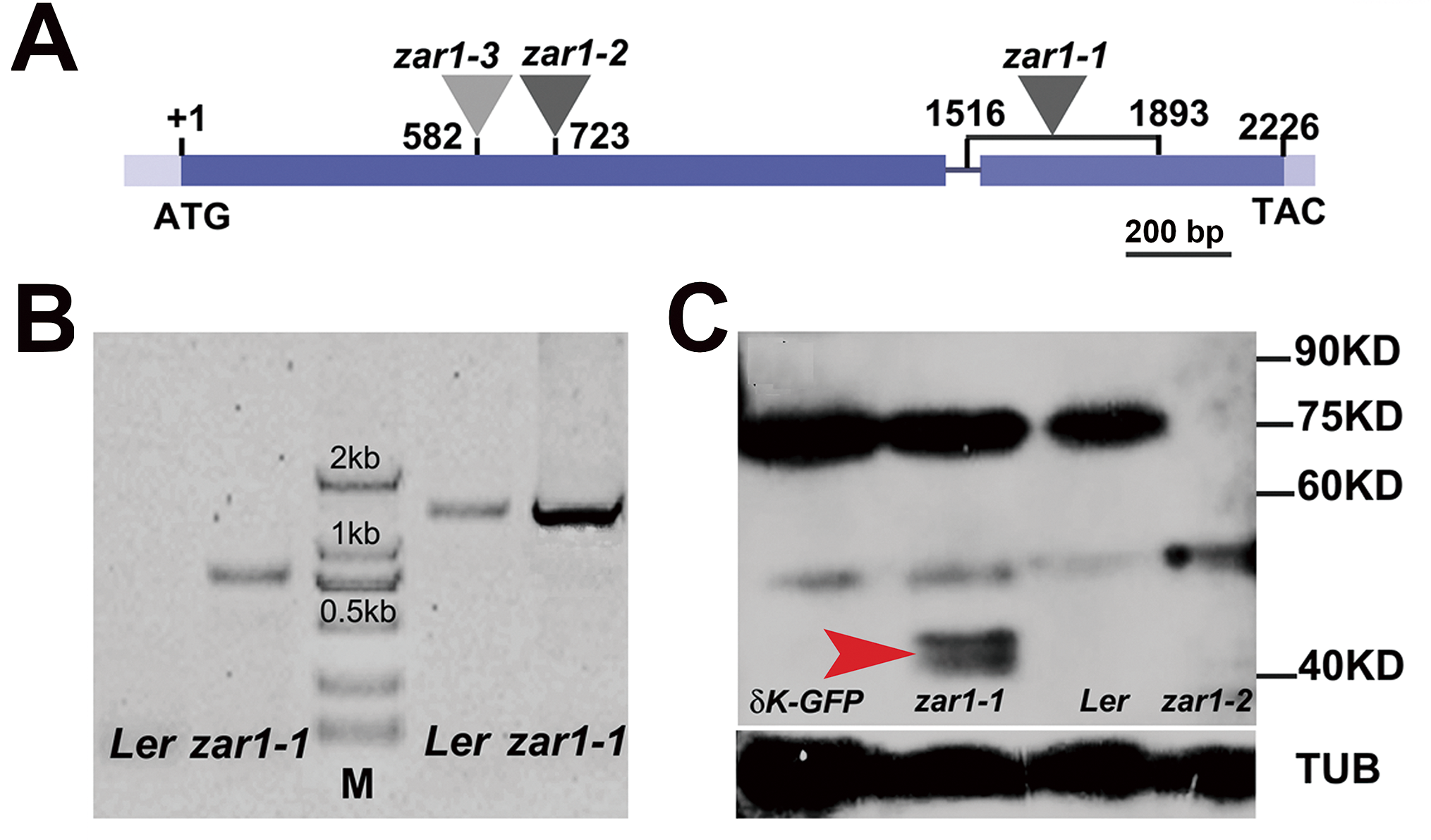

Supplement: S6 Fig — (A) ZAR1 gene structure with insertion sites indicated. (B) RT-PCR analysis showing a truncated mRNA in zar1-1+/-. (C) Western blot showing the truncated ZAR1 protein (arrowhead) in zar1-1+/-. TUB6 was used as loading control. (TIF) [file pgen.1005933.s006.tif]

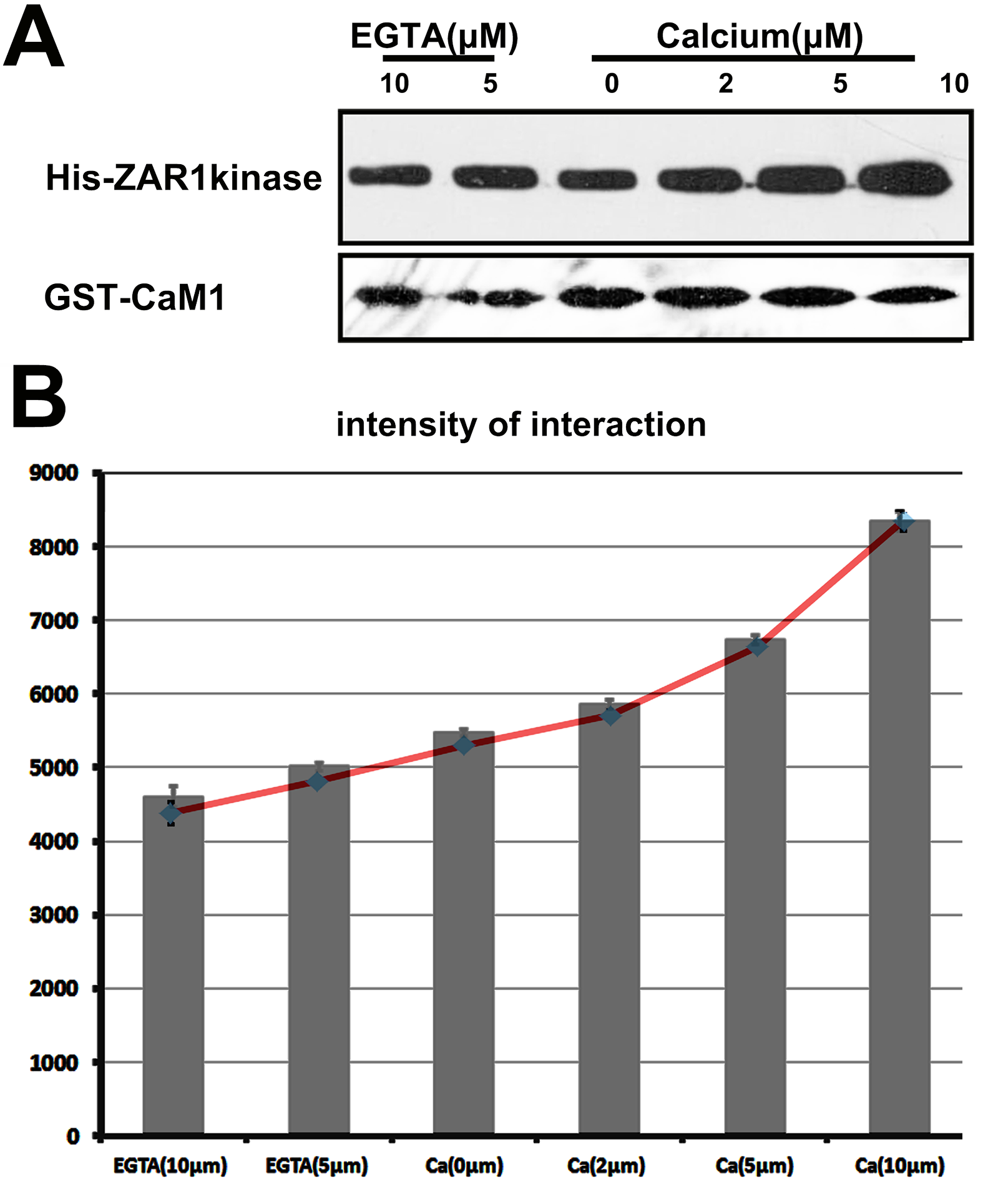

Supplement: S7 Fig — The interaction of ZAR1 and CaM1 is shown in pull-down assay (A). The statistical analysis indicates that the interaction is enhanced with the increasing concentration of calcium (B). (TIF) [file pgen.1005933.s007.tif]

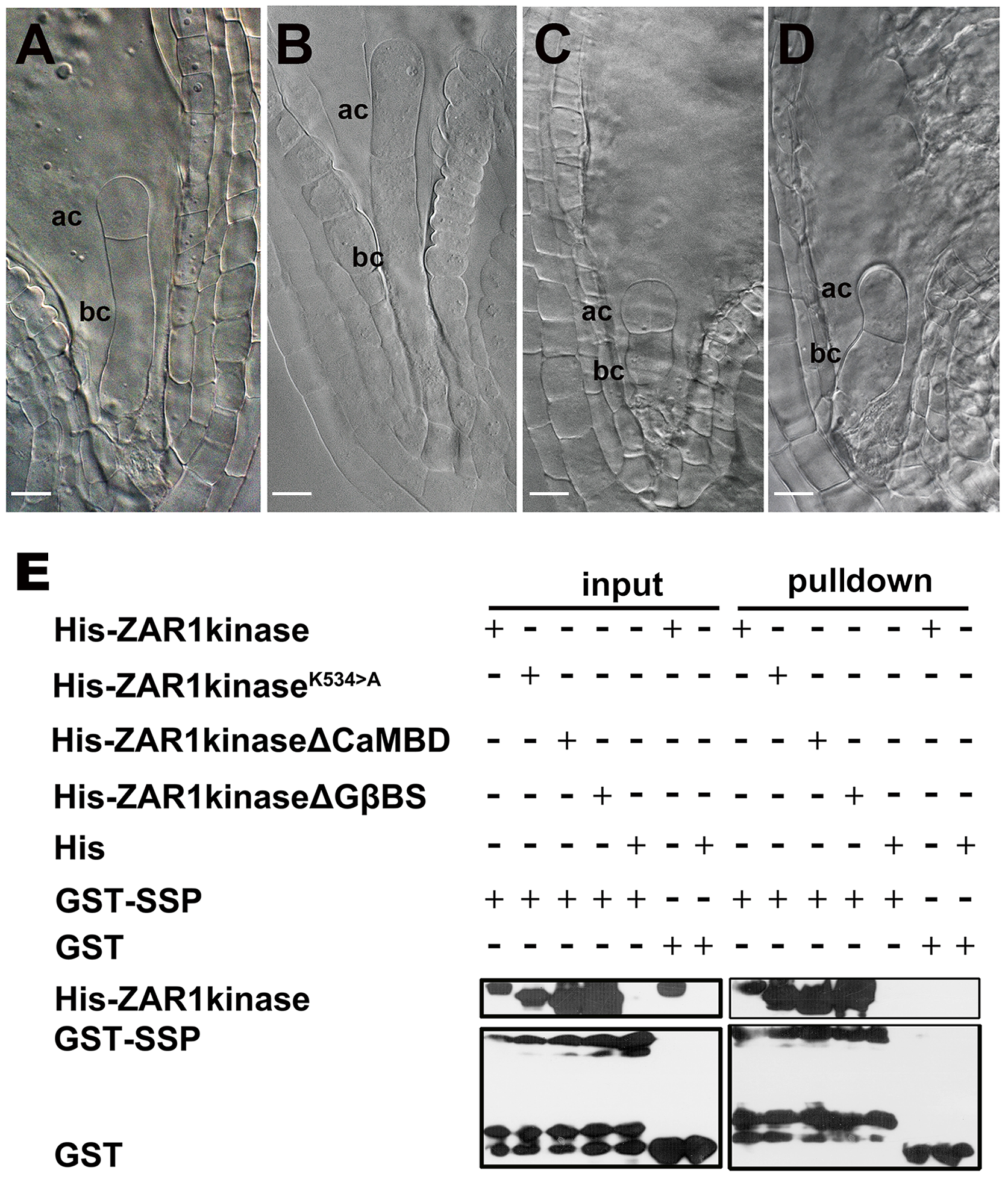

Supplement: S8 Fig — The symmetric division in zar1-1 (B), ssp (C) and zar1-1/ssp (D), compared to the wild type (A). The immature seeds of ssp-/-/zar1-1+/- plants (D) showed very similar phenotype to ssp-/- mutant (C). ac, apical cell; bc, basal cell. Bars = 10 μm. (E) Pull-down assay showing interaction between His-tagged ZAR1 kinase domain with GST-tagged SSP protein. (TIF) [file pgen.1005933.s008.tif]
