# Supplementary material for: The Arabidopsis Receptor Kinase ZAR1 Is Required for Zygote Asymmetric Division and Its Daughter Cell Fate
Source: PLoS Genet. 2016 Mar 25;12(3):e1005933. doi: 10.1371/journal.pgen.1005933 (PMC4807781; doi:10.1371/journal.pgen.1005933)
Supplement: S1 Table — (DOCX) [file pgen.1005933.s009.docx]

**S1 Table. List of Primers**

| AD1 | 5’-NTCGA(G/C)T(G/C)G(A/T)GTT-3’ |
| --- | --- |
| AD2 | 5’-NGTCGA(G/C)(A/T)GANA(A/T)GAA-3’ |
| AD3 | 5’-(A/T)CAGNTG(A/T)TNGTNCTG-3’ |
| AD4 | 5’-TG(A/T)GNAG(G/C)ANCA(G/C)AGA-3’ |
| AD5 | 5’-AG(A/T)GNAG(A/T)ANCA(A/T)AGG-3’ |
| AD6 | 5’-(G/C)TTGNTA(G/C)TNCTNTGC-3’ |
| AD7 | 5’-(A/T)GTGNAG(A/T)ANCANAGA-3’ |
| Ds3-1 | 5’-ACCCGACCGGATCGTATCGGT-3’ |
| Ds3-2 | 5’-CGATTACCGTATTTATCCCGTTC-3’ |
| Ds3-3 | 5’-GTATTTATCCCGTTCGTTTTCGT-3’ |
| Ds3-4 | 5’-CCGTCCCGCAAGTTAAATATG-3’ |
| Ds5-1 | 5’-ACGGTCGGGAAACTAGCTCTAC-3’ |
| Ds5-2 | 5’-CCGTTTTGTATATCCCGTTTCCGT-3’ |
| Ds5-3 | 5’-TACCTCGGGTTCGAAATCGAT-3’ |
| Ds5-4 | 5’-TACGATAACGGTCGGTACGG-3’ |
| ZAR1-confirm-F | 5’-ATTGAAGGACCTTTGCCAAGGATG-3’ |
| ZAR1-confirm-R | 5’-CTATTCACGCAACTGATT-3’ |
| ZAR1-com-F | 5’-GGGGTACCCCATGTTGGCCTCGCTGATCATCTTC-3’ |
| ZAR1-com-R | 5’-AACTGCAGAATCAATCGCCGGCCACGGGTAATCT-3’ |
| ZAR1-△K-GFP-F | 5’-AACTGCAGAATGTTTCTCTCGGTTTCGTCTCCTCC-3’ |
| ZAR1-△K-GFP-R | 5’- CGGGATCCCGTGGAGGGCGGTTGCGAGGTTAC-3’ |
| ZAR1-3UTR-F | 5’-GGGGTACCCCGACATCTAGAGATTGCACATAAAGC-3’ |
| ZAR1-3UTR-R | 5’- CGGAATTCCGGTCTGAGTTCTCTTTGGTCTACAGA-3’ |
| GFP-F | 5’-GGGGTACCATGGTGAGCAAGGGCGAGGAGCT-3’ |
| GFP-R | 5’-CGGGATCCTTACTTGTACAGCTCGTCCATGC-3’ |
| ZAR1-kinase-his-F | 5’-GGGAATTCAAGTTCTGCGCTTGTAACCGCGAGAACCAA-3’ |
| ZAR1-kinase-his-R | 5’-GCGTCGACATCGCCGGCCACGGGTAATCTGTCGAGCGT-3’ |
| CaM1-GST-F | 5’-CGGGATCCATGGCGGATCAACTCACTGACGAAC-3’ |
| CaM1-GST-R | 5’-ACGCGTCGACTCACTTAGCCATCATAATCTTGACA-3’ |
| CaM8-GST-F | 5’-GGAATTCCGATGGAAGAAACAGCACTGACAAAAG-3’ |
| CaM8-GST-R | 5’-ACGCGTCGACTCAGTCAATGTTGATCATCATCTTG-3’ |
| Deta-CaM1bs-F | 5’-GCTCTTGTGATGGTGATCCGTTTGGATGA-3’ |
| Deta-CaM1bs-R | 5’-GAACCAATTCGGAGTCGAATCCGAAACGCC-3’ |
| Deta-Gβbs-F | 5’-GCTCTTGTGATGGTGATCCGTTTGGATGA-3’ |
| Deta-Gβbs-R | 5’-TCATCCAAACGGATCACCATCACAAGAGC-3’ |
| K-A-ZAR1-kinase-F | 5’- TACGTCCATGGAGATCTCGCGCCAAGCAACATTCTCATT-3’ |
| K-A-ZAR1-kinase-R | 5’-AATGAGAATGTTGCTTGGCGCGAGATCTCCATGGACGTA-3’ |
| AGB1-MBP-F | 5’- CGGGATCCATGTCTGTCTCCGAGCTCAAAGAAC-3’ |
| AGB1-MBP-R | 5’-GTCGACAATCACTCTCCTGTGTCCTCCAAAC-3’ |
| ZAR1-GFP-F | 5’-CGAGCTCATGTTGGCCTCGCTGATCATCTTCGTT-3’ |
| ZAR1-GFP-R | 5’-GGGGTACCATCGCCGGCCACGGGTAATCTGTCGAG-3’ |
| ZAR1-Q-F | 5’-GGATTGCTACAGGGCTTGTT-3’ |
| ZAR1-Q-R | 5’-GAATGTTGCTTGGCTTGAGA-3’ |
| deltaK-ZAR1-Q-F | 5’-GGATTGCTACAGGGCTTGTT-3’ |
| deltaK-ZAR2-Q-R | 5’ –GAATGTTGCTTGGCTTGAGA-3’ |
| ZAR1LRR-Q-F | 5’-CCAAGAAAAGGGCTTCTGAG-3’ |
| ZAR1LRR-Q-F | 5’-TTTAGCAGCTCCTCCAGGTT-3’ |
| ACTIN2/8-Q-F | 5'-GGTAACATTGTGCTCAGTGGTGG-3' |
| ACTIN2/8-Q-R | 5'-AACGACCTTAATCTTCATGCTGC-3' |
| ZAR1-BiFC-CE-F | 5’- GGACTAGTATGTTGGCCTCGCTGATCATCTTCG-3’ |
| ZAR1-BiFC-CE-R | 5’-CCGCTCGAGATCGCCGGCCACGGGTAATCTGTCG-3’ |
| AGB1-BiFC-NE-F | 5’- GCTCTAGAATGTCTGTCTCCGAGCTCAAAGAAC-3’ |
| AGB1-BiFC-NE-R | 5’-GGGGTACCAATCACTCTCCTGTGTCCTCCAAAC-3’ |
| CaM1-BiFC-NE-F | 5’-GCTCTAGAATGGCGGATCAACTCACTGACGAAC-3’ |
| CaM1-BiFC-NE-R | 5’- GGGGTACCCTTAGCCATCATAATCTTGACAAAC-3’ |
| AGB1-RFP-F | 5’- GCTCTAGAATGTCTGTCTCCGAGCTCAAAGAAC-3’ |
| AGB1-RFP-R | 5’-GGGGTACCAATCACTCTCCTGTGTCCTCCAAAC-3’ |
| CaM1-RFP-F | 5’-GCTCTAGAATGGCGGATCAACTCACTGACGAAC-3’ |
| CaM1-RFP-R | 5’- GGGGTACCCTTAGCCATCATAATCTTGACAAAC-3’ |
| ZAR1-FLAG-F | 5’-GGGGTACCATGTTGGCCTCGCTGATCATCTTCG-3’ |
| ZAR1-FLAG-R | 5’-GGTTCGTTATCGCCGGCCACGGGTAATCTGTCG-3’ |
| CaM1-HA-F | 5’-GGGGTACCATGGCGGATCAACTCACTGACGAAC-3’ |
| CaM1-HA-R | 5’-GCGTCGACCTTAGCCATCATAATCTTGACAAAC-3’ |
| AGB1-HA-F | 5’- GGGGTACCATGTCTGTCTCCGAGCTCAAAGAAC-3’ |
| AGB1-HA-R | 5’- GCGTCGACAATCACTCTCCTGTGTCCTCCAAAC-3’ |
| CaMRLK kinase-MBP-F | 5’-GCTCTAGAATGCGCCGGAGAATGAAATCGGCGAGAAC-3’ |
| CaMRLK kinase-MBP-R | 5’-ACGCGTCGACACGTGAAACCGTACGTATGTCTTTAAG-3’ |
| ZAR1-GFP-F | 5’-CTTGATATCGAATTCCTGCAGCCATGTTGGCCTCGCTGATCAT |
| ZAR1-GFP-R | 5’-CCTTGCTCACCATTCTAGAACTAGTATCGCCGGCCACGGGTAATC-3’ |
| AGB1-mCherry-F | 5’-CTTGATATCGAATTCCTGCAGCCATGTCTGTCTCCGAGCTCAA-3’ |
| AGB1-mCherry-R | 5’-CGCCCTTGCTCACTCTAGAACTAGTAATCACTCTCCTGTGTCCTC-3’ |
