# Supplementary material for: The Arabidopsis Receptor Kinase ZAR1 Is Required for Zygote Asymmetric Division and Its Daughter Cell Fate
Source: PLoS Genet. 2016 Mar 25;12(3):e1005933. doi: 10.1371/journal.pgen.1005933 (PMC4807781; doi:10.1371/journal.pgen.1005933)
Supplement: S2 Table — (DOCX) [file pgen.1005933.s010.docx]

**S2 Table. Expression analysis of *pWOX8gΔ:NLS-vYFP_3_***

**in *zar1-2* and *agb1-2***

| genotype of mother plant | n | signal in suspensor(%) | signal in embryo(%) |
| --- | --- | --- | --- |
| 2-4 cell stage |  |  |  |
| wild type | **212** | **100.0** | **3.8** |
| *zar1-2* | **306** | **98.4** | **64.7** |
| *agb1-2* | **287** | **98.3** | **58.5** |
| 8 cell stage |  |  |  |
| wild type | **238** | **100.0** | **0.0** |
| *zar1-2* | **311** | **97.8** | **40.2** |
| *agb1-2* | **265** | **97.7** | **28.3** |
| late globular stage |  |  |  |
| wild type | **236** | **98.7** | **0.0** |
| *zar1-2* | **346** | **98.1** | **13.0** |
| *agb1-2* | **292** | **97.3** | **6.8** |
