# Supplementary material for: The Arabidopsis Receptor Kinase ZAR1 Is Required for Zygote Asymmetric Division and Its Daughter Cell Fate
Source: PLoS Genet. 2016 Mar 25;12(3):e1005933. doi: 10.1371/journal.pgen.1005933 (PMC4807781; doi:10.1371/journal.pgen.1005933)
Supplement: S1 Text — (DOCX) [file pgen.1005933.s011.docx]

**S1 Text. Supporting materials and methods**

Analysis of truncation in *zar1-1^+/-^*

The RT-PCR analysis of ZAR1 expression in *zar1-1^+/-^* and wild type (S2 Fig B) shows transcript of *DS-ZAR1* (primers using Ds5-4 and ZAR1-confirm-F) in *zar1-1^+/-^* and transcript of *ZAR1* in the wild-type L*er* and *zar1-1^+/-^* (using ZAR1-confirm-F and ZAR1-com-R). The total RNA was extracted from the inflorescence of L*er* and *zar1-1^+/-^*. The total protein extracted from the inflorescence and young siliques was detected with ZAR1-specific LRR motif antibody, TUB6 was used as loading control. The antibody to LRR motif (shown as following) was prepared by (Saier, Tianjin).

Sequence of LRR antigen:

VHDDPTGSLNNWNSSDENACSWNGVTCKELRVVSLSIPRKNLYGSLPSSLGFLSSLRHLNLRSNRFYGSLPIQLFHLQGLQSLVLYGNSFDGSLSEEIGKLKLLQTLDLSQNLFNGSLPLSILQCNRLKTLDVSRNNLSG
